# Identifying a spatial scale for the analysis of residential burglary: An empirical framework based on point pattern analysis

**DOI:** 10.1371/journal.pone.0264718

**Published:** 2022-02-28

**Authors:** Mohammed A. Alazawi, Shiguo Jiang, Steven F. Messner

**Affiliations:** 1 Department of Information Science, University at Albany, State University of New York, Albany, NY, United States of America; 2 Department of Geography and Planning, University at Albany, State University of New York, Albany, NY, United States of America; 3 Department of Sociology, University at Albany, State University of New York, Albany, NY, United States of America; National Taiwan University, TAIWAN

## Abstract

A key issue in the spatial and temporal analysis of residential burglary is the choice of scale: spatial patterns might differ appreciably for different time periods and vary across geographic units of analysis. Based on point pattern analysis of burglary incidents in Columbus, Ohio during a 9-year period, this study develops an empirical framework to identify a useful spatial scale and its dependence on temporal aggregation. Our analysis reveals that residential burglary in Columbus clusters at a characteristic scale of 2.2 km. An ANOVA test shows no significant impact of temporal aggregation on spatial scale of clustering. This study demonstrates the value of point pattern analysis in identifying a scale for the analysis of crime patterns. Furthermore, the characteristic scale of clustering determined using our method has great potential applications: (1) it can reflect the spatial environment of criminogenic processes and thus be used to define the spatial boundary for place-based policing; (2) it can serve as a candidate for the bandwidth (search radius) for hot spot policing; (3) its independence of temporal aggregation implies that police officials need not be concerned about the shifting sizes of risk-areas depending on the time of the year.

## Introduction

In spatial analysis of crime such as residential burglary, a key issue is the choice of the spatial scale of analysis. In areal data analysis, the spatial scale of analysis refers to the unit of analysis. In the literature [see 1 for a brief review], studies have adopted different units/scales varying from the macro level (nation, state, county or city) to meso level (neighborhood, census tract), to the micro level (street addresses or street segments). The spatial patterns of crime may vary across geographic units of analysis [2–5], which is the well-known Modifiable Areal Unit Problem [MAUP; 6–8]. Therefore, it is difficult to compare or generalize across different studies due to the issues of the ecological fallacy and the atomistic fallacy [3]. For example, statistical inferences about the nature of larger geographic units (groups) are not necessarily applicable to smaller geographic units due to the issue of the ecological fallacy [3,5,9]. Similarly, statistical inferences based on smaller geographic units cannot be directly applied to larger geographic units due to the issue of the atomistic fallacy [3,10,11].

Another issue is the choice of the most useful temporal scale. In its finest scale, crime incidents are recorded at the resolution of the minute. In practice, crime data are usually aggregated based on a certain time interval, mostly on a yearly basis to be consistent with the temporal scale of other covariates with available data such as socio-economic status (SES). Some studies have also aggregated multiple years of crime data to minimize yearly fluctuations [12,13]. However, the existence of seasonality [14–16], repeat and near-repeat victimization [17–19], and periodic change of guardianship for homes [20,21] can all affect the temporal patterns of residential burglary. The seasonality observed for property crimes and specifically for burglary has been confirmed by many other studies [15,22–25] and has mostly been explained with reference to routine activity theory. Andresen and Malleson [14] argued that, due to the seasonal patterns and related distinct spatial patterns, analysis based on yearly aggregated crime rates and census data may not be suitable for inference. Studies of repeat and near-repeat victimization reveal that victimization recurs quickly to the same victim or to targets with similar characteristics or situation [19,26]. The time intervals between recurrence can be as short as one week but are mostly within 4–6 weeks [21,27,28]. Periodic change of guardianship for homes refers to the difference of guardianship for weekday/weekend and day/night wherein the least degree of guardianship usually occurs during the daytime of weekdays [20,29]. The interaction among seasonality, periodic change of guardianship, and repeat/near repeat victimization creates complex spatio-temporal patterns that may be masked by the yearly aggregated data.

Previous studies have made significant contributions in searching for a useful scale of analysis. Criminologists have examined crime concentration at various spatial levels (e.g., street segment, neighborhood, and district) and found that the majority of the variability can be attributed to street segments [30–32], confirming the importance of crime analysis at microscale [1,33–36]. Some scholars also investigated the in(stability) of residential burglary patterns on street segments and found that burglary point patterns exhibit a moderate to high degree of spatial stability over time [37]. Despite the current movement within criminology towards analyzing crime at finer spatial scales, some scholars have recently revealed otherwise different results. For example, Malleson et al. [38] found that the choice of scale varies with the types of crime, the number of events, and the degree of clustering, and Ramos et al. [39] revealed that finer is not necessarily better in the micro-analysis of crime, and that units coarser than street segments might be better.

In this study, we borrow ideas from ecological and biological research and use *L* function to identify the scale of analysis for crime patterns. In the biological literature, different forms of *K* function (*L* function) have been used to investigate the spatial organization (random, clustering, or regularity) of molecules [40,41], to identify the domain size (cluster size) of micro-organization [42–44] or degree of clustering [45], and the change of spatial point pattern over time [46]. This study uses residential burglary as an example and extends the ideas from the above biological research in pursuit of two closely related objectives:

1. to develop an empirical framework that can facilitate the selection of the spatial scale of analysis for residential burglary;
2. to examine the impact of temporal aggregation on the spatial scale identified in the first objective.

It is important to acknowledge at the onset that selecting the scale of analysis depends upon the objectives of the research. For example, research on the influence of welfare policies on crime rates in the U. S. has typically used the U. S. states as the units of analysis. This is justified on the grounds that state-level agencies determine and administer the welfare policies which apply to the respective residents [47,48]. Similarly, a macro-sociological theory such as Institutional Anomie Theory [49] has stimulated a good deal of research at the level of nation-states, which makes sense given that the core variables in the theory reflect features of societies, e.g., the interrelationships among social institutions and dominant cultural values. We also acknowledge the practical benefits of crime hot spot policing demonstrated in the literature [50–55].

In our study, the search for the spatial scale seeks to identify the geographic scale that captures faithfully the clustering of incidents in the data. This is thus an inductive, empirical approach to identify meaningful areal units. Our approach is predicated on the assumption that observed spatial clustering/spatial dependence of residential burglary is likely to indicate social processes that do in fact operate at the corresponding geographic scale. The results of our study shed light on the selection of appropriate units for areal studies, thus addressing the modifiable areal unit problem (MAUP).

## Data and methods

### Study area and data

Our study area is based on data for the city of Columbus, Ohio, which is the capital of Ohio and the county seat of Franklin County. From the U.S. Census TIGER website, we obtained the boundary file of Columbus, which is a little messy and has many isolated holes or dangling areas. To reduce the edge effect [56–58] due to complex boundaries, we determined the study area with a balance between selecting as large an expanse of the Columbus city as possible and as simple/compact a boundary as possible.

**Fig 1:**
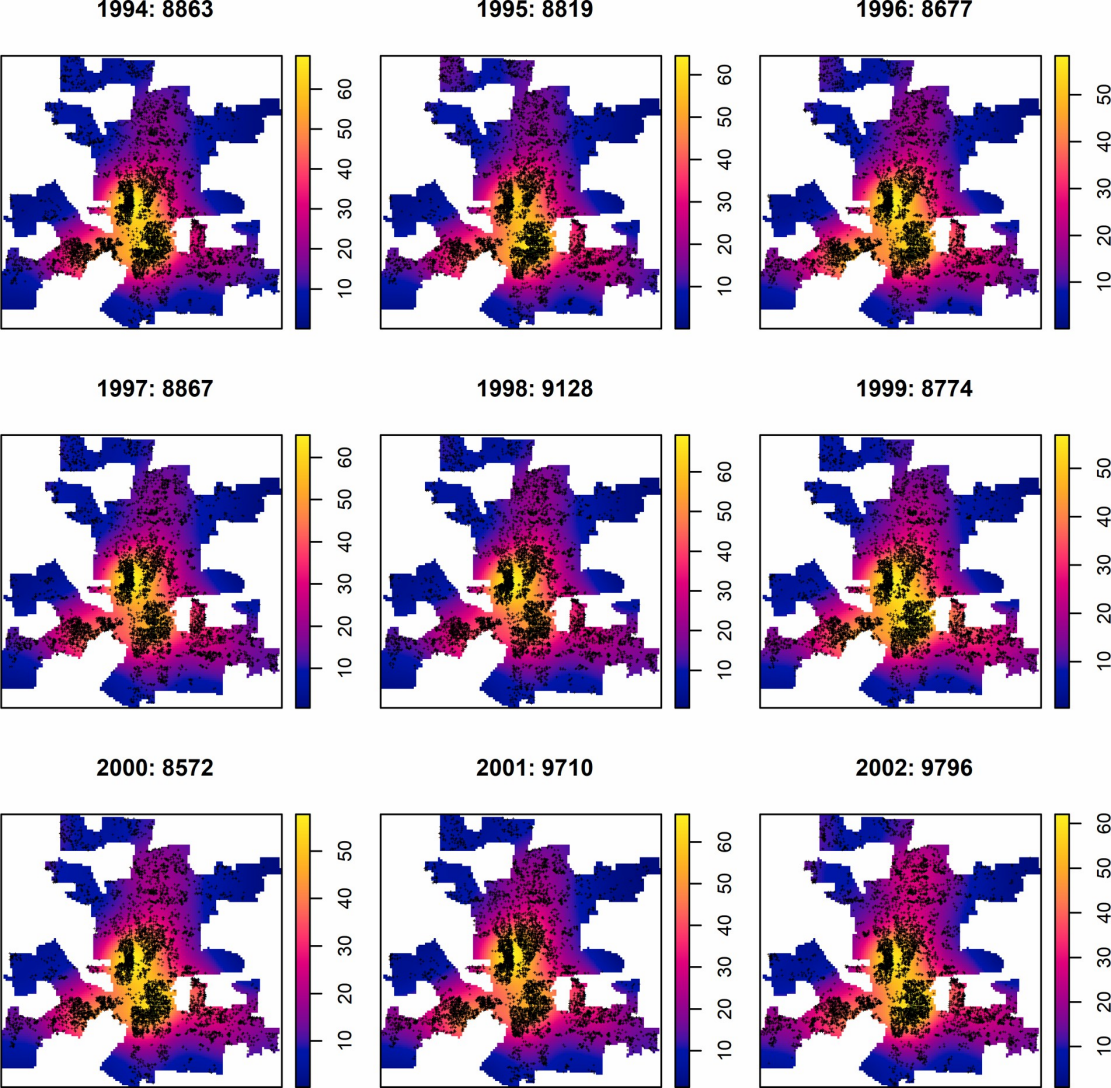
Residential burglary (represented with black +) overlaid with kernel density map indicating hotspots around downtown Columbus during 1994–2002. In each subplot, the year and the number of offenses are separated by “:”; For example, in the first subplot on the top left, “1994: 8863” indicates that there were 8863 residential burglary offences in year 1994. The unit of the density map is one residential burglary per km^2^.

We obtained residential burglary data (TXT files) for the years 1994–2002 from the Columbus Division of Police. We cleaned and geocoded the data to points using ESRI ArcGIS with a 96% match rate, which far exceeded the minimum acceptable match rate (i.e., 85%) of geocoding proposed by Ratcliffe [59]. The mapped spatial point patterns of residential burglary during 1994–2002 are shown in Fig 1, where the number of offenses appears in each subplot after the year. The yearly number of offenses during 1994–2002 range from 8572 to 9796, with an average of 9023. Other ancillary data include: (1) the block data and parcel data of Franklin County, Ohio, all obtained from Franklin County Auditor; (2) the boundaries of census tracts and zip codes obtained from the 2000 U.S. Census-TIGER/Line Shapefiles; (3) the boundaries of the neighborhoods obtained from Google My Maps [60], (4) the boundaries of Columbus communities obtained from City of Columbus Department of Technology [61].

To investigate the impact of temporal aggregation on the spatial scale of the crime pattern, we classify residential burglary based on five temporal levels, with 1+4+12+2+7 = 26 temporal scales and 26 x 9 = 234 spatial point patterns for the nine years (Table 1). The descriptive statistics for all the 234 spatial point patterns are presented in the Supplementary Data (S1 Table).

**Table 1:** Temporal scale of point pattern analysis.

Level	Scale	Number of temporal scale per year	Number of years	Total number of point patterns
**Annual**	1994–2002	1	9	9
**Season**	Spring-Winter	4	9	36
**Month**	January-December	12	9	108
**Workday**	Weekday/Weekend	2	9	18
**Day of week**	Monday-Sunday	7	9	63
**Grant total**		26	9	234

### Point pattern analysis

We investigate how each residential burglary offense (point) is located in space compared to other residential burglary offenses (points) at various temporal scales. We conduct this analysis using Ripley’s *K* function [57,62; for simplicity, K function hereafter], a statistic based on the pairwise distances between events, i.e., burglary offences for this study. Based on the intensity and the distance distribution of the events, *K* function can detect if the point patterns are completely random, clustered, or regular (inhibition between events). Point pattern analysis using *K* function has been widely used in statistics and geography and has gained increasing use in criminology in recent years [e.g., 63–65]. One specific feature of *K* function analysis that has been relatively neglected or underreported in previous studies is the scale of clusters, which can be used to guide the determination of scale for crime studies.

#### Homogeneous K function

Ripley’s *K* function [62] measures the within-pattern point interactions and can be used to create a summary graph of cumulative crimes indicating the cluster pattern at multiple scales. Specifically, *K* function can reveal the spatial scales (distances) where significant spatially clustered point patterns occur. The original Ripley’s *K*-function assumes stationary point processes with a constant intensity. Consider a stationary spatial point process *U* = {*u*_1_,…,*u*_*n*_}. So, *K* value within a Euclidian radius distance of *h* for the observed number of points *N* over a study area *A* is estimated as:

$$\hat{K}(h)=\frac{1}{\left|A\right|}\sum_{i=1}^{N}\sum_{j=1,j\neq i}^{N}\frac{e_{ij}I(d_{ij}\leq h)}{\lambda^{2}}$$ (1)

where *λ* is the intensity of the point process that can be estimated by *λ* = *N*(*A*)/|*A*|, which is constant throughout the study area *A*, and |*A*| is the area of the study area *A*, *N*(*A*) is the number of events in study area *A*; *d*_*ij*_ is the distance between location of point *i* and location of point *j* that fall inside a circle of radius *h*, so, *I* (*d*_*ij*_ *< h*) is the indicator function where:

$$I(d_{ij}\leq h)=\{\begin{array}{l} 1,\;\mathrm{If}\;d_{ij}\leq h\\ 0,\;\mathrm{If}\;d_{ij}>h \end{array}$$ (2)

*e*_*ij*_ is an edge correction term to remove the bias introduced by the edge of study area *A* [57]. Point process under *K*-function is assumed as homogenous Poisson Process, also called complete spatial randomness (CSR), where intensity is homogeneous throughout a study area.

#### Inhomogeneous K function

Exploratory analyses of the first order intensity maps (see the maps in Fig 1 for some examples) indicate the presence of a large-scale (global) trend from the downtown area outwards. This large-scale trend is likely due to the spatial variation of the population at risk, i.e., the inhomogeneous distribution of residential properties [66]. This means that applying the original *K*-function could be misleading because the significant spatial dependence (clustering) could be a result of the first order intensity (due to inhomogeneous point process) rather than the second order spatial dependence that occurs within the point pattern. For example, the spatial point pattern of residential burglary could show a significant spatial dependence within certain spatial scales due to a small-scale variation of the intensity resulting from the variation of the spatial population of parcels rather than the real spatial interaction within the crime point pattern. Here, the population parcels could be the hidden covariate that resulted in the spatial trend of intensity in the residential burglary.

Therefore, when testing the null hypothesis of complete spatial randomness, it is necessary to consider the spatial trend effects shown in the corresponding intensity map of Fig 1 [67,68]. In other words, the point patterns of residential burglary in Columbus can be modeled by a nonstationary inhomogeneous Poisson process (IPP) with non-constant intensity [69,70]. To do so, we separate the spatial trend and re-weight the first order intensity by using inhomogeneous *K*-function [71]. Such separation is necessary to avoid the confounding between intensity and within-pattern interaction [72,73].

The inhomogeneous *K*-function (*K*_inhom_) is an extension of Ripley’s homogeneous *K*-function to an inhomogeneous point process, where spatial dependence and associated spatial scales are examined with non-constant intensities throughout a study area. Under *K*_inhom_ function, the spatial varying intensities and the spatial trend are adjusted by an intensity function $\hat{\lambda }(u)$. Consider a non-stationary spatial point process *U* = {*u*_1_,…,*u*_*n*_} with an intensity function $\hat{\lambda }(u)$, the *K*_inhom_ is estimated as [71,74]:

$${\hat{K}}_{\mathrm{inhom}}(h)=\frac{1}{\left|A\right|}\sum_{i=1}^{N}\sum_{j=1,j\neq i}^{N}\frac{e_{ij}I(d_{ij}\leq h)}{\hat{\lambda }(u_{i})\hat{\lambda }(u_{j})}$$ (3)

where |*A*|, *d*_*ij*_, *I*(*d*_*ij*_ *< h*), and *e*_*ij*_ are defined as before; $\hat{\lambda }(u_{i})$ and $\hat{\lambda }(u_{j})$ are the intensity function *λ*(*u*) at point *u*_*i*_ and *u*_*j*_, respectively, which are estimated using the method in next section. To stabilize the variance of *K* function and for visualization purpose, we use *L* function which is a centered linear transformation of *K* function [66,74–76]:

$${\hat{L}}_{\mathrm{inhom}}(h)=\sqrt{{\hat{K}}_{\mathrm{inhom}}(h)/\pi }-h$$ (4)

To determine the significance of the *L* function, we construct a simulation envelope using the Monte Carlo test [57,77,78], which only requires a relatively small number of simulations to achieve high accuracy [73]. In this study, we conduct 39 Monte Carlo simulations, resulting in a significance level of *α* = 2/(1+39) = 0.05 according to Baddeley et al. [73]. Again, the border correction method developed by Ripley [79] is used to remedy the possible edge effect bias (*e*_*ij*_). The border correction strategy is preferable for large point data set to allow faster computation. In addition, compared to other edge effect strategies and methods, the border correction estimate is consistent and approximately unbiased [73].

#### Modeling the spatial trend

This intensity function $\hat{\lambda }(u)$ can be estimated non-parametrically by using kernel smoothing estimators or nearest-neighbor estimators [71,80,81] or parametrically by applying a parametric model (e.g., a log-linear model). To investigate the general spatial patterns of residential burglary, we estimate the first order intensity of each point pattern using kernel smoothing with a bandwidth of 2000 m. Fig 1 shows the intensity maps for each year of 1994–2002. To avoid bias due to edge effect and to obtain better statistical performance, we use the edge correction method proposed by Diggle [80]. The estimated intensity map, with relatively large bandwidth (i.e., 2000 m here), reveals the general spatial trend of the point patterns [69,80].

To further characterize the first order intensity used for the within-pattern interaction analysis, a parametric model is preferred for two main reasons. First, the non-parametric estimation through kernel smoothing is using the same spatial point pattern data, which can lead to substantial bias in the estimate of the inhomogeneous *K*-function, which is more responsive to local fluctuations in the data [73]. Second, the non-parametric estimation has the assumption that the scale of the first order is larger than that of the second order [67].

**Fig 2:**
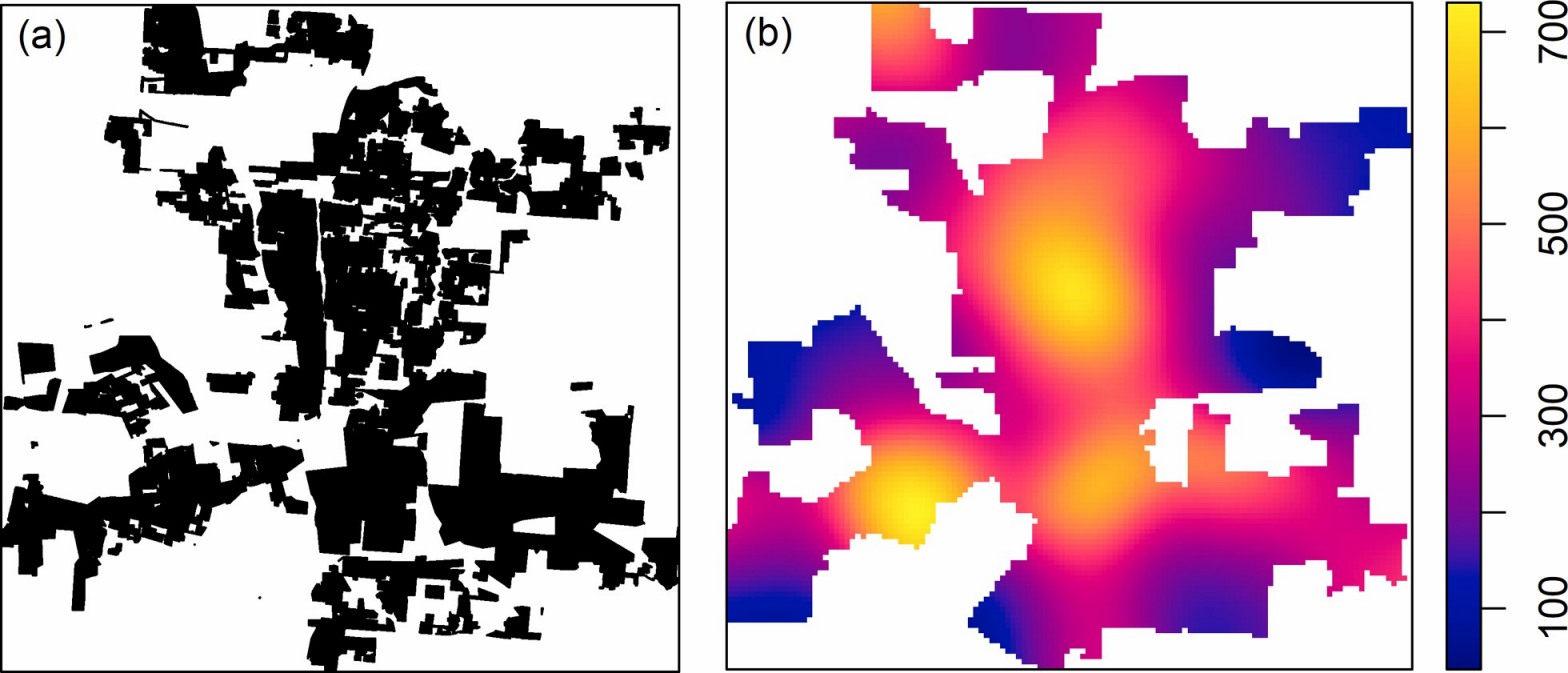
Spatial covariates for modeling the spatial trend. a–residential area, b–parcel density (unit: One parcel per km^2^).

For this study, the global trend for the point pattern of residential burglary can be estimated with a log-linear additive model [73]:

$$\hat{\lambda }(u)=\exp\{\beta_{0}+\beta_{1}x_{1}(u)+\beta_{2}x_{2}(u)+\ldots +\beta_{p}x_{p}(u)\}$$ (5)

where *β*_0_, *β*_1_, *β*_2_,⋯,*β*_*p*_ are the parameters to be estimated; *x*_1_(*u*), *x*_2_(*u*),⋯,*x*_*p*_(*u*) are the covariates at location *u*. The spatial covariates used in Eq 5 should be the populations at risk, where we include two variables (Fig 2): residential area (RA) and parcel density (PD).

By definition, residential burglary should only happen in the residential area. However, due to mixed parcel/land use, there are a significant number of residential burglary incidents in areas classified as non-residential. To characterize this variation, we include residential area as a dummy variable in all our candidate models. The spatial covariates are converted to raster images to link with the point pattern through the model in the R environment. The residential areas are represented as a dummy variable: 1 for residential areas and 0 for non- residential areas. The raster image of parcel density is estimated using kernel density estimation.

The models are validated using the relative intensity, *r*(*u*), which measures the agreement between the true intensity *λ*(*u*) and the estimated intensity $\hat{\lambda }(u)$ [73]. The relative intensity is defined as $r(u)=\lambda (u)/\hat{\lambda }(u)$. The closer *r*(*u*) to 1, the better the model. In practice, *r*(*u*) is estimated by kernel smoothing [73]:

$$\hat{r}(u)=\frac{1}{e(u)}\sum_{i}\frac{1}{\hat{\lambda }(u_{i})}k(u-u_{i})$$ (6)

where *k* is a smoothing kernel and *e*(*u*) is the edge correction factor.

#### Spatial scale of interaction

**Fig 3:**
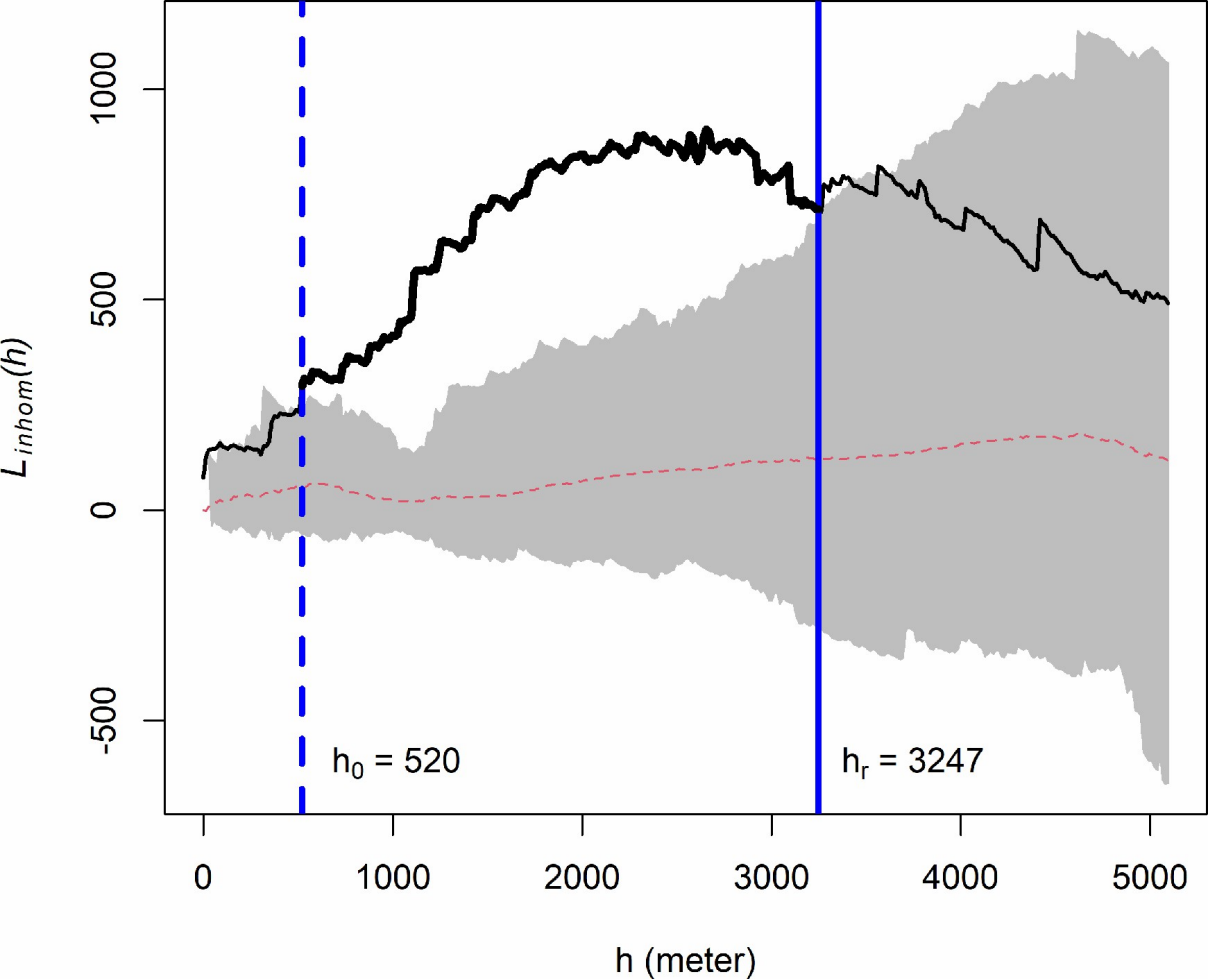
An example inhomogeneous *L* function, assuming inhomogeneous Poisson process (IPP). The black line (including the thick segment in the middle) is the estimated *L* function from the data; the red dashed line is the theoretical *L* function (null model) for IPP; the shaded area indicates the simulation envelope constructed with 39 Monte Carlo simulations of the fitted IPP model. The blue dashed line indicates the minimum scale of interaction (*h*_0_); the blue solid line indicates the range of interaction (*h*_*r*_).

The *K* function reflects the number of events separated by certain distance *h*. The *L* function is the standardized *K* function, indicating the standardized strength of interaction. Fig 3 shows an example of an estimated *L* function (thick black line) and simulation envelope (gray shaded area).

For a clustered point pattern, the inhomogeneous *L* function starts inside the simulation envelope and increases with distance *h*. At a certain distance, inhomogeneous *L* function rises consistently above the simulation envelope. After a certain distance, the inhomogeneous *L* function decreases until below the simulation envelope (Fig 3). Here we define three scales of interaction.

- Minimum Scale of Interaction (*h*_0_): the distance where the *L* function starts to increase above the simulation envelope as indicated by the blue dashed line in Fig 3 where *h*_0_ = 520 meters.
- Range of Interaction (*h*_*r*_): the distance where the *L* function decreases below the upper envelope as indicated by the blue solid line in Fig 3 where *h*_*r*_ = 3247 meters. *h*_*r*_ ≤ *H*, where *H* is the maximum distance between events in the study area.
- Characteristic Scale of Clustering (*h*_*c*_): the weighted average scale of interaction defined as,

$$h_{c}=\frac{\sum_{h_{n}\leq h_{i}\leq h_{r}}L_{i}h_{i}}{\sum_{h_{n}\leq h_{i}\leq h_{r}}L_{i}}$$ (7)

where *L*_*i*_ is the estimated *L* value for *h*_*i*_, *h*_0_≤*h*_*i*_≤*h*_*r*_, i.e., *h*_*c*_ is estimated based on the thick black line between the blue dashed line and blue solid line.

Under some conditions, particularly for the point patterns at annual aggregation, the *L* function stays above the envelope all through the *h* values. In such case, *h*_*r*_ is calculated as the maximum distance (*H*) between events in the study area.

### Dependence of spatial scale on temporal aggregation

To examine the impact of temporal aggregation on the spatial scale of clustering, we analyze spatial point patterns with various temporal aggregations. In other words, we subset the point pattern data by different temporal scales, and then conduct point pattern analysis using Ripley’s *K* function to detect the spatial scales of clustering. As discussed in Table 1 above, we analyze 234 spatial point patterns with various temporal aggregations, resulting in 234 sets of *L* functions and spatial scales of interaction. We use a one-way ANOVA test to compare the difference and significance of the characteristic scale of clustering among the 234-point patterns.

Most of the analyses in this paper are carried out using R [82]. Specifically, the point pattern analysis (*K* function and *L* function) is implemented using functions from the spatstat package [73].

## Results

### Spatial trend of residential burglary

**Fig 4:**
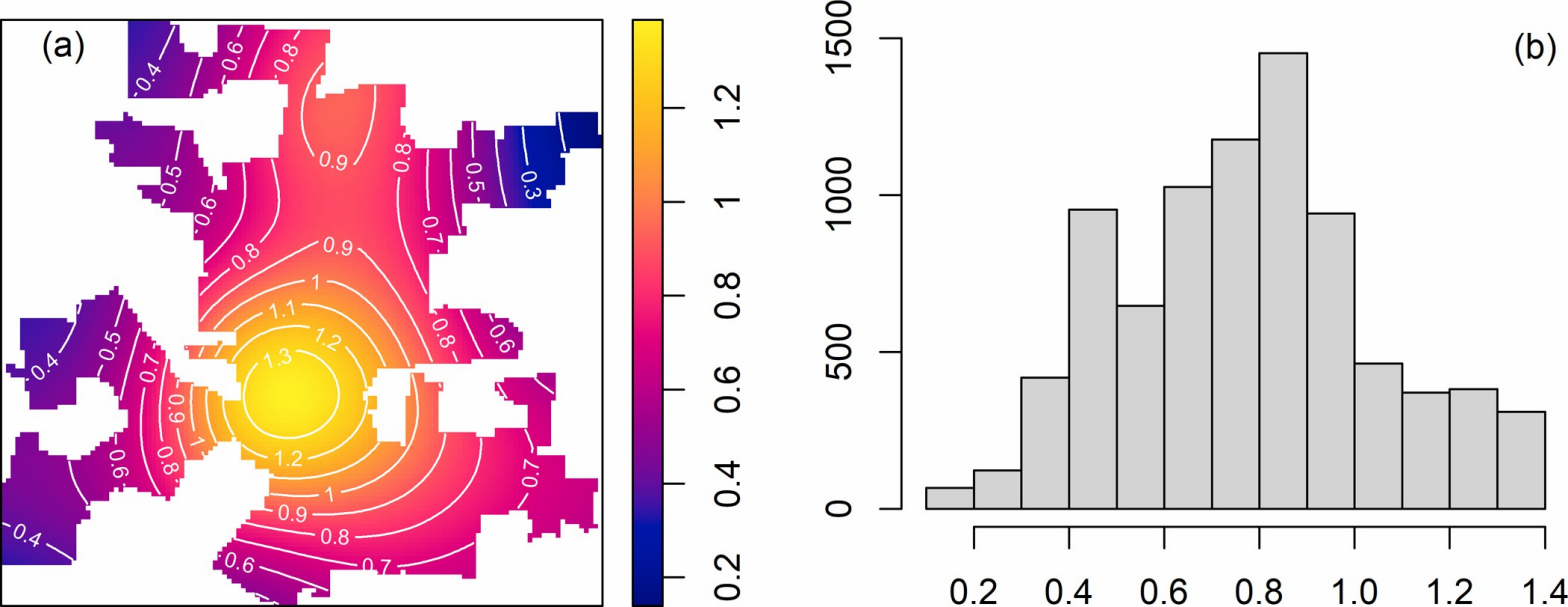
Estimated relative intensity.

The estimated spatial trend model is: $\hat{\lambda }(u)=\exp\{-2.83+2.63\mathrm{RA}(u)+0.34\mathrm{PD}(u)\}$, where RA is the residential area and PD is the parcel density as shown in Fig 2. All the parameters, *β*_0_, *β*_1_, *β*_2_, are significant at the level *α* = 0.001. The map and histogram of estimated relative intensity are shown in Fig 4.

**Fig 5:**
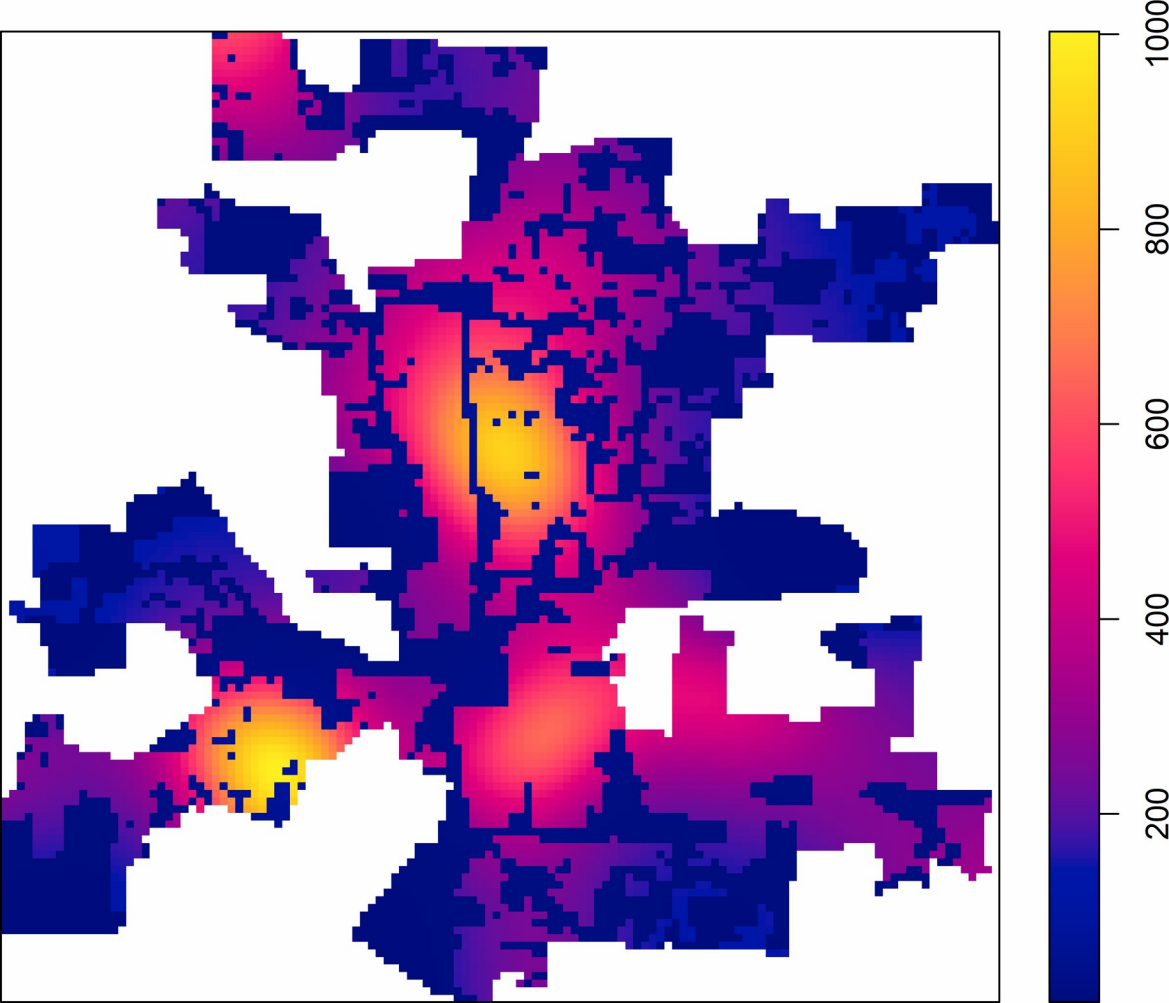
Fitted intensity map for mapped point pattern (all residential burglary during 1994–2002). Unit: One residential burglary incident per km^2^.

The range of the relative intensity is concentrated around 0.7–1. Fig 5 shows the fitted intensity map for all residential burglaries during 1994–2002. The map indicates that the spatial trend of residential burglary is well captured by the spatial covariates. The estimated parameters of the model indicate that the intensity of residential burglary is *e*^2.63^≈14 times higher in residential areas than non-residential areas (i.e., mixed use parcel/areas). For each additional parcel per km^2^, the residential burglary incidents increase by (*e*^0.34^−1)×100%≈40.5%.

### Spatial scale of clustering

**Fig 6:**
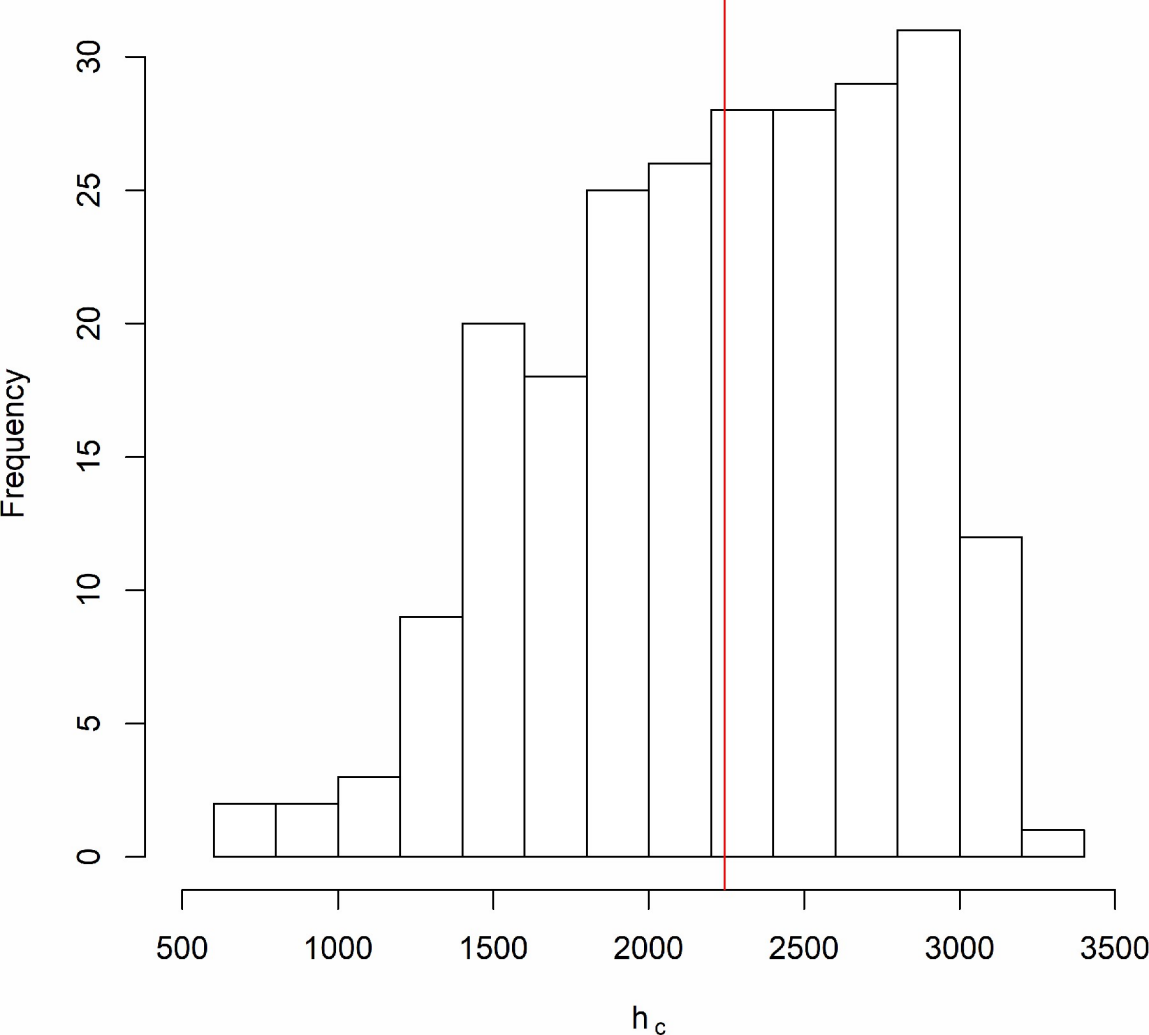
The distribution of the characteristic scale of clustering (*h*_*c*_) across the 234-point patterns. The red vertical line highlights the grand mean of *h*_*c*_ (2243 m).

For all the 234 point patterns, the inhomogeneous *L* functions stay above the simulation envelopes for at least a certain distance. In other words, all the point patterns are significantly clustered. For each of the 234 point patterns, we extracted the minimum scale of interaction (*h*_0_), the range of interaction (*h*_*r*_), and the inhomogeneous *L* function values (*L*_*i*_) associated with distances *h*_*i*_ (*h*_0_ ≤ *h*_*i*_ ≤ *h*_*r*_). For the example *L* function shown in Fig 3, the extracted *L*_*i*_ and *h*_*i*_ are shown in S2 Table of the Supplementary Data. Using Eq 7, we calculated the characteristic scale of clustering (*h*_*c*_) (Table 2), which varies across the temporal scales with a grand mean of 2243 meters. As shown by the histogram (Fig 6), the distribution of *h*_*c*_ across values most concentrated between 1500 m and 3000 m.

**Table 2:** The characteristic scale of clustering (*h*_*c*_) for the 234-spatial point patterns.

Year	Annual	Spring	Summer	Fall	Winter	Jan	Feb	March	April	May	Jun	Jul	Aug	Sep	Oct	Nov	Dec	Weekday	Weekend	Mon	Tue	Wed	Thu	Fri	Sat	Sun
**1994**	1987	1385	2357	2582	2959	2326	3101	2387	984	1852	1554	1914	2869	2149	2288	2869	3013	2266	2552	2411	2770	2159	2282	2204	2724	1925
**1995**	2102	2607	2207	2648	1835	1678	2008	2270	1691	3083	2956	2492	1574	1882	1784	2870	1518	2356	2504	2932	1892	2034	1812	1908	2340	2004
**1996**	2160	2716	2779	2725	2707	1628	1401	2389	2275	2178	3008	2751	1786	2763	2291	2692	2763	2468	2712	2951	2348	2849	2748	2481	2682	2091
**1997**	1992	2735	2575	1789	2653	3167	2916	2445	1124	2895	1928	2423	1326	1627	1640	2824	1976	2189	2801	1705	2151	2952	1539	2596	3007	1873
**1998**	2007	2496	2619	2914	2626	1958	1824	2685	2488	1769	1861	2321	2621	1741	1670	3164	2751	2325	2392	2448	2787	2210	2286	2892	1557	2130
**1999**	2106	1488	2818	2093	2921	2925	2845	1168	1297	1567	1753	3082	2725	2965	1915	1693	1468	2434	2388	2840	2550	1869	1546	2958	2399	2461
**2000**	2037	2844	2159	2254	1456	927	614	692	3056	2507	1596	2446	1942	1585	2374	1574	2585	1967	2875	2734	1545	1951	2197	1423	2851	2062
**2001**	1965	2870	2903	2880	1638	1576	1227	1979	2237	3208	2560	2231	2642	2756	3088	1657	1064	2319	2530	2621	2893	2829	2503	3024	2861	1304
**2002**	1943	2796	2158	1662	2524	2545	1456	2198	2494	1514	1695	1579	1912	1328	1261	2255	2474	2128	2004	3015	2051	2086	1321	2599	1304	2175
**Descriptive Statistics**																										
**min**	1943	1385	2158	1662	1456	927	614	692	984	1514	1554	1579	1326	1328	1261	1574	1064	1967	2004	1705	1545	1869	1321	1423	1304	1304
**max**	2160	2870	2903	2914	2959	3167	3101	2685	3056	3208	3008	3082	2869	2965	3088	3164	3013	2468	2875	3015	2893	2952	2748	3024	3007	2461
**mean**	2033	2438	2509	2394	2369	2081	1932	2024	1960	2286	2101	2360	2155	2088	2035	2400	2179	2273	2529	2629	2332	2327	2026	2454	2414	2003
**sd**	70	546	279	438	537	678	812	622	675	622	547	412	533	566	508	582	654	148	245	385	433	402	462	498	566	294

Note: The grand descriptive statistics are as follows: min(*h*_*c*_) = 614; max(*h*_*c*_) = 3208; mean(*h*_*c*_) = 2243; sd(*h*_*c*_) = 546.

### Dependence of spatial scale on temporal aggregation

The ANOVA test among the spatial patterns of residential burglary based on the temporal scale indicates that changing temporal scale has no significant impacts on the spatial scale of interaction (*h*_*c*_) (Table 3). In other words, the spatial scales of interaction are relatively stable across various temporal scales.

**Table 3:** ANOVA test for the characteristic scale of clustering among the spatial point patterns based on temporal aggregations.

	Df	Sum Sq	Mean Sq	F value	Pr (>F)
**Characteristic scale of clustering**	25	9048921	361957	1.239	0.209
**Residuals**	208	60781093	292217		

## Summary and discussion

### Spatial trend of residential burglary and population at risk

Our spatial trend model captured most of the first order intensity of residential burglary and thus served the purpose of separating the global trend and local within pattern variation of residential burglary. As noted, this separation is essential to investigate spatial dependence. For Columbus Ohio, compared to mixed parcel/land use, the residential area has a much higher risk of residential burglary as expected. Parcel density, as an indicator of the population at risk, captures the spatial patterns of residential burglary very well.

### Spatial scale of clustering

**Fig 7:**
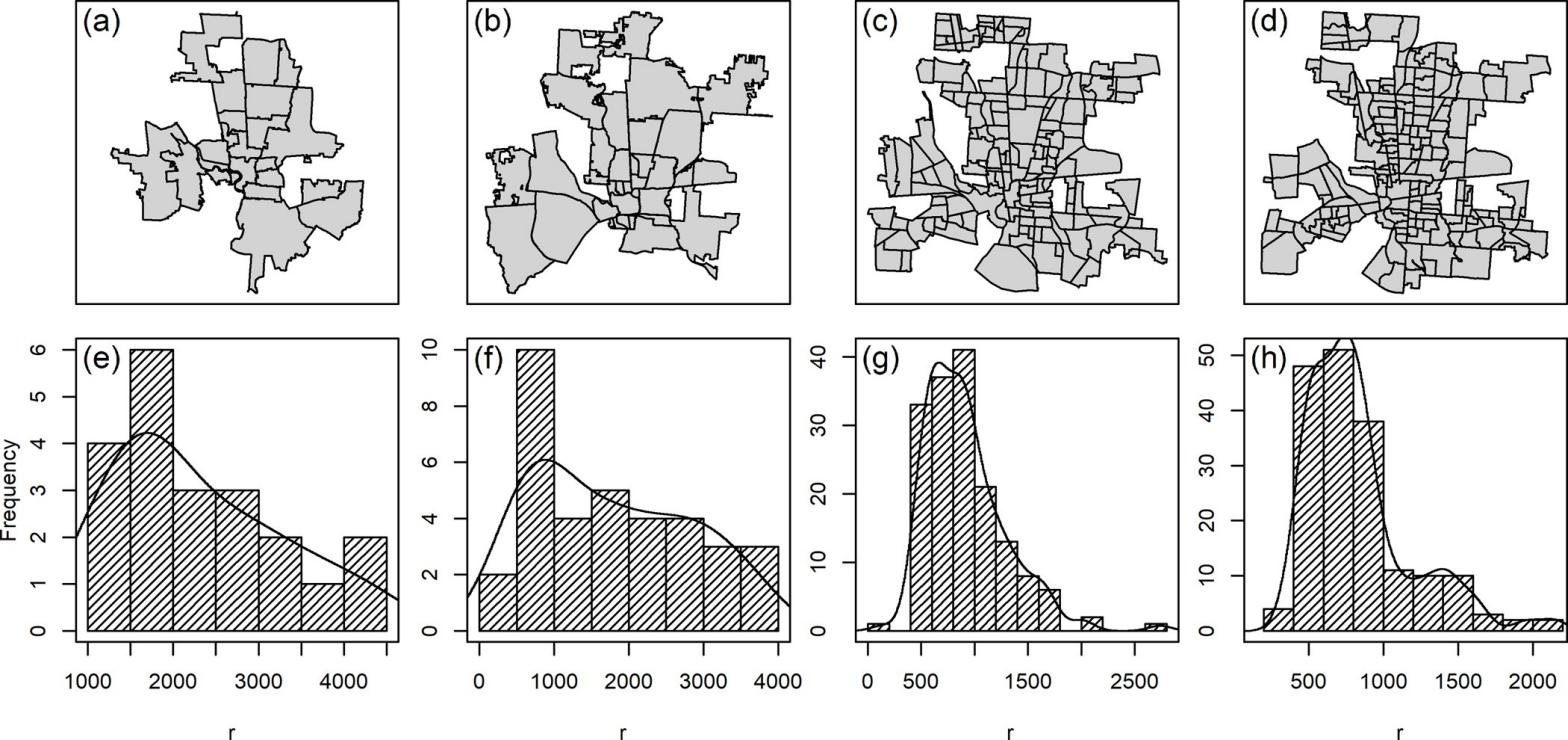
The boundaries and radius distribution for four area units: zip codes (a and e), communities (b and f), neighborhoods (c and g), and census tracts (d and h). For the boundaries of communities and zip codes, we included only the units that most of their areas fall within the study area and excluded those that most boundaries extend beyond the study area.

The characteristic scale of clustering (*h*_*c*_ = 2243 m) is the radius of the geographic unit where the spatial variation of the residential burglary is captured. For a given residential burglary event, the chance of finding another residential burglary event within 2243 meters is higher than beyond 2243 meters. We compared the characteristic scale of clustering with the equivalent radius of four geographic units in the study area: census tracts, neighborhoods, communities, and zip codes (Table 4). The average sizes of the equivalent radius for census tracts and neighborhoods are 829 meters and 911 meters, respectively, which are less than half the characteristic scale of clustering (2243 meters). The average of the equivalent radius for zip codes (2290 meters) is the closest to the characteristic scale of clustering. However, the spread distribution shows that the radius of the zip code is positively skewed with the maximum of 4472 meters, resulting in several units that are much larger than the average of the characteristic scale of clustering (Fig 7(E)). This can result in overestimating the geographic area of the processes of residential burglary and the cluster of residential burglary, as will be discussed later in this section.

**Table 4:** Descriptive statistics of the equivalent radius for zip code, community, neighborhood, census tract, and the characteristic scale of clustering (*h*_*c*_) in the study area.

Geographic Unit	No. of Units	Radius				
		Min	Max	Median	Mean	SD
**Zip code**	21	1029	4472	2007	2290	1000
**Community**	35	336	3698	1730	1776	1074
**Neighborhood**	163	338	2734	859	911	376
**Census tract**	179	136	2142	748	829	354
***h*_*c*_**	234*	614	3208	2286	2243	547

* Represents the number of spatial scales, i.e., 234 spatial point patterns. The equivalent radius is calculated as $\sqrt{\mathrm{area}/\pi }$, i.e., the radius of the equivalent circles.

**Fig 8:**
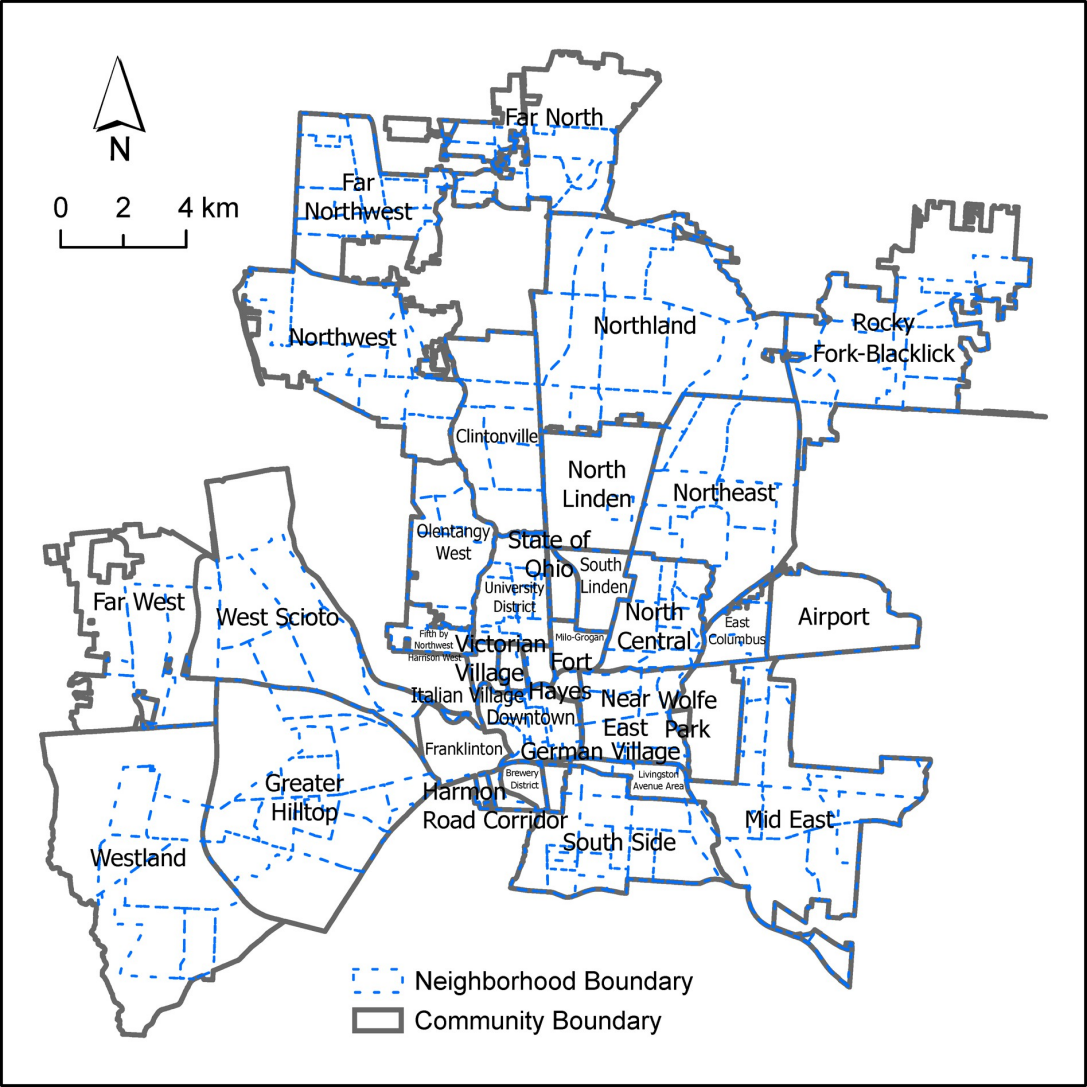
The configuration of communities and neighborhoods in the study area.

The characteristic scale of clustering reflects, to a large extent, the size and distribution of commonly understood communities in the study area (Table 4, and Fig 7(F)), which are composed of several neighborhoods and used by the city of Columbus for planning and reporting purposes (Fig 8). The community units have the mean and medium equivalent radius of 1776 meters and 1730 meters, respectively, with the maximum radius of 3698 meters, which is the closest to the maximum characteristic scale of clustering (3208 meters). As shown in (Fig 7(F)), the radius for 83% of the community units are equal to or less than the average characteristic scale of clustering, and the radius for the rest of the units fall within the range of the characteristic scale of clustering, suggesting the community unit is the most suitable unit of analysis among the other geographic units.

Our empirical, inductive approach yields useful information to help guide conventional analyses of crime rates in urban communities. For areal data analysis, crime data are commonly aggregated into areal units so that crime rates for these units can be correlated with social-economic factors. One important consideration for the selection of the size of such areal units is that they conform to the area defined by the characteristic scale of clustering.

1. If analysts choose an areal unit larger than $\pi h_{c}^{2}$, they would be aggregating significant clusters with non-clustered areas, i.e., smoothing residential burglary rates, leading to the loss of information.
2. If they choose an areal unit smaller than $\pi h_{c}^{2}$, there is chance of dividing a significant cluster into multiple area units.

Given that the clusters do not necessarily coincide with areal units, there is always the chance of dividing significant clusters into multiple areal units even when choosing one with size equal to $\pi h_{c}^{2}$. This is not particularly problematic because each areal unit tends to be homogeneous, without the loss of information. In other words, analysts are on solid ground when implementing an areal unit that comes as close to our criterion given the available data. For the residential burglary of Columbus, $\pi h_{c}^{2}=15.8\;{\mathrm{km}}^{2}$, which is about the mean size of communities in the study area. These results suggest that the community is a geographic unit that is particularly well suited for the spatial analysis of residential burglary in Columbus.

### Impact of temporal scale on the spatial scale of interaction

We found no significant impact of temporal aggregation on the spatial scale of interaction. Changing the temporal aggregation level could result in a changing frequency of crime or a different spatial pattern in terms of location. However, the results indicate that the spatial scale and geographic level of significant–clustered pattern (the size of that spatial pattern) does not depend on the temporal scale. This stability across various temporal scales suggests strong social or spatial processes that operate within that range of spatial scales. It is important to mention that the large variation in the number of crime incidents across the 234-point patterns (mean = 1735, standard deviation = 1919, see S1 Table in the Supplementary Data for details) does not necessarily impact the values of the characteristic scale of clustering (*h*_*c*_).

Our results regarding the independence of the spatial scale with respect to the temporal scale are consistent with previous spatio-temporal studies where spatial dependence is more significant and has a larger influence on crime patterns than spatio-temporal interaction [83,84]. It also implies that police officials need not be concerned about shifting sizes of risk-areas with the time of the year.

### Further implications

Our paper addresses the well-known Modifiable Areal Unit Problem (MAUP), whereby different boundaries and spatial scales result in different visual representations and hot spots of crime [85]. As shown in previous studies [86], burglary is related to both neighborhood-level and target-level attributes. The MAUP is a potential concern for researchers who are interested in causal studies of crime (e.g., residential burglary here) and the effects of explanatory variables [87]. Thus, this paper provides a preliminary attempt and framework that can help researchers deal with the issue of geographic aggregation level.

Another implication is related to the issue of using fixed pre-existing geographic units with non-overlapping boundaries as the units of analysis. Lee and colleagues [88,89] argued that such geographic units (e.g., census tracts) do not reflect the actual area for the community concept (e.g., neighborhood) or the spatial environment of criminogenic processes. They therefore proposed a tract-free approach across egocentric local environments of varying size (radius). Hipp and colleagues [12,90] similarly argued that neighborhoods defined based on nonoverlapping boundaries (such as block groups or tracts) are fundamentally flawed in investigating the spatial processes of crime. They proposed a new definition of neighborhoods labelled “egohoods”, which are conceptualized as waves washing across the surface of cities using overlapping boundaries. The new measures proposed by Lee, Hipp and colleagues [12,88–90] used (weighted) averages of data within circles of various radii centered on the target block. The characteristic scale of clustering *h*_*c*_ derived using our method might serve as a promising candidate for the radius put forth by Lee, Hipp and colleagues.

Moreover, our approach provides important information to those who conduct hot spot analyses, especially those involving bandwidth selection such as in kernel density estimation, where the characteristic scale of clustering can be used to facilitate the determination of the bandwidth. Our study can be combined with hot spot analyses to help effective policing: Our method reveals the characteristic scale of clusters, while the hot spot analyses reveal the location of clusters. The characteristic scale of clustering (*h*_*c*_) also complements the hot spot policing strategy by serving as a *radius search* for residential burglary hot spots. For the case of residential burglary in Columbus, Ohio, our results can guide police agencies in their efforts to control and prevent residential burglary by extending their interventions to 2.2 km from the highly focused spots of residential burglary.

Our study also supports previous research in geographic profiling that has documented processes underlying target selection for residential burglary. Summers et al. [91] found that burglars have a consistent pattern of semi-radial movement in different directions from their home. The characteristic scale of clustering in our study may represent such a radius of the circle wherein burglars commute to commit burglaries. Our results for the characteristic scale of clustering (2.2 km) in residential burglary is comparable to the travel distances of burglars revealed in previous studies, such as 2.6 km in Rhodes & Conley [92], 2.8 km in White [93], 2.6-3km in Wiles & Costello [94]. Similarly, in a study of burglary in the Hague, the Netherlands, Bernasco [95] found that the majority (about 90%) of the solitary burglars travel a distance of 1–4 km to commit crime, with an estimated mean of 2.6 km.

Our analytic framework can be used to address MAUP by a wide range of applications that use areal units for spatial analysis, such as disease outbreak, public health issues, and health inequalities [96,97], environmental risk analysis [98], social and population analysis [99], spatial politics analysis [100]. Moreover, the framework can be used to address the Modifiable Temporal Unit Problem (MTUP) that is associated with MAUP [101,102].

### Limitations and future study

Our spatial trend model (Eq 5) mainly relies on the population at risk (parcels) and the polygons that determine the residential areas (dummy variable). This model can be improved to capture more accurately the first order effects (intensity of residential burglary) by including more covariates that contribute to the large-scale variation of residential burglary.

Although our findings provide valuable insight about the spatial pattern of residential burglary and the selection of an appropriate spatial scale of analysis, the theoretical rationale and social spatial processes that generate such spatial patterns are not clear. In addition, the appropriate scale presented here applies to residential burglary in Columbus, Ohio. It may vary across different offenses and differ for different cities. Thus, one potential and useful extension of this study is to apply the basic analytic framework to different types of crime across different cities. This comparison can help develop a theoretical framework that can explain the variation or uniformity of spatial patterns for different types of crime. In the case where the appropriate scale resulting from point pattern analysis does not overlay well with census units, area based interpolation can be applied to generate areal data at the appropriate scale [103–105].

It is also important to investigate the possible presence of micro space-time interaction that can change the location of clusters over time. This stability or instability of cluster locations reveal how long the crime clusters persist at the determined spatial scale of analysis, which has important implications for deploying resources from the police force.

## Conclusion

In this study, we applied spatial point pattern analysis to characterize the within pattern interaction of residential burglary incidents and its dependence on temporal variability. The inhomogeneous *L* functions was used to determine the characteristic scale of clustering, which can serve to identify a reasonable spatial scale of analysis. Residential burglary mostly clusters at a spatial scale of 15.8 km^2^, which is about the size of communities in our study area. This finding suggests that the community is well suited for spatial analysis of residential burglary in Columbus, Ohio. We found no significant variation of the spatial scales of clustering for spatial patterns aggregated at different temporal scales. Thus, it is reasonable to use a yearly temporal aggregation level for spatial analyses of residential burglary.

Overall, we call attention to two main implications from this study. First, it has policy implications given that the characteristic scale of clustering can be used to define the spatial boundary for place-based policing. Second, it can lay the foundation for theoretical explanations of the social processes that operate within the characteristic scale of clustering. In areal data analysis of crime risk, we suggest that crime data and socioeconomic factors be aggregated at a spatial scale that comes close to our criterion given the available data.

## Supporting information

S1 Table Counts (number of incidents) and distances (km) between residential burglary incidents for all the 234 point patterns. (DOCX)

S2 Table *L*_*i*_ and *h*_*i*_ for Fig 3: *h*_0_≤*h*_*i*_≤*h*_*r*_, *h*_0_ = 519.5, *h*_*r*_ = 3247.2. (DOCX)

S1 Data (ZIP)
