# Supplementary material for: Identifying a spatial scale for the analysis of residential burglary: An empirical framework based on point pattern analysis
Source: PLoS One. 2022 Feb 28;17(2):e0264718. doi: 10.1371/journal.pone.0264718 (PMC8884495; doi:10.1371/journal.pone.0264718)
Supplement: S1 Table — (DOCX) [file pone.0264718.s001.docx]

**Table S1** **Counts (number of incidents) and distances (km) between residential burglary incidents for all the 234 point patterns**

| **Temporal Scale** | | **Year** | **Counts** | **Distance Between Incidents** | | |
| --- | --- | --- | --- | --- | --- | --- |
|  |  |  |  | **Mean** | **Range** | **Sd** |
| Annual |  | 1994 | 8863 | 9.1 | 0-34.0 | 5.3 |
|  |  | 1995 | 8819 | 9.6 | 0-34.2 | 5.5 |
|  |  | 1996 | 8677 | 9.6 | 0-34.1 | 5.5 |
|  |  | 1997 | 8867 | 9.5 | 0-34.1 | 5.4 |
|  |  | 1998 | 9128 | 9.5 | 0-34.1 | 5.4 |
|  |  | 1999 | 8774 | 9.6 | 0-34.1 | 5.4 |
|  |  | 2000 | 8572 | 9.6 | 0-34.0 | 5.4 |
|  |  | 2001 | 9710 | 9.6 | 0-34.0 | 5.4 |
|  |  | 2002 | 9796 | 10.1 | 0-36.0 | 5.6 |
| Season | Spring | 1994 | 1755 | 9.6 | 0-33.2 | 5.4 |
|  |  | 1995 | 1812 | 9.5 | 0-33.6 | 5.6 |
|  |  | 1996 | 1843 | 9.6 | 0-33.8 | 5.5 |
|  |  | 1997 | 1708 | 9.6 | 0-33.2 | 5.4 |
|  |  | 1998 | 1924 | 9.4 | 0-33.3 | 5.3 |
|  |  | 1999 | 1851 | 9.8 | 0-33.4 | 5.5 |
|  |  | 2000 | 1896 | 9.6 | 0-33.5 | 5.4 |
|  |  | 2001 | 1985 | 9.6 | 0-33.3 | 5.4 |
|  |  | 2002 | 2026 | 10.5 | 0-33.1 | 5.8 |
|  | Summer | 1994 | 3286 | 8.8 | 0-33.8 | 5.2 |
|  |  | 1995 | 3213 | 9.5 | 0-34.2 | 5.6 |
|  |  | 1996 | 3066 | 9.5 | 0-34.1 | 5.4 |
|  |  | 1997 | 3139 | 9.5 | 0-33.8 | 5.4 |
|  |  | 1998 | 3311 | 9.2 | 0-34.1 | 5.3 |
|  |  | 1999 | 3202 | 9.7 | 0-33.8 | 5.4 |
|  |  | 2000 | 3115 | 9.4 | 0-33.9 | 5.4 |
|  |  | 2001 | 3438 | 9.7 | 0-33.9 | 5.4 |
|  |  | 2002 | 3436 | 9.7 | 0-32.9 | 5.5 |
|  | Fall | 1994 | 1853 | 9.1 | 0-33.2 | 5.2 |
|  |  | 1995 | 1619 | 9.8 | 0-33.3 | 5.5 |
|  |  | 1996 | 1639 | 9.8 | 0-31.8 | 5.4 |
|  |  | 1997 | 1774 | 9.4 | 0-34.1 | 5.5 |
|  |  | 1998 | 1726 | 10.1 | 0-34.0 | 5.6 |
|  |  | 1999 | 1813 | 9.5 | 0-33.2 | 5.4 |
|  |  | 2000 | 1553 | 9.7 | 0-32.7 | 5.4 |
|  |  | 2001 | 2004 | 9.4 | 0-33.6 | 5.3 |
|  |  | 2002 | 1963 | 10.3 | 0-33.7 | 5.7 |
|  | Winter | 1994 | 1969 | 9.0 | 0-32.5 | 5.2 |
|  |  | 1995 | 2175 | 9.6 | 0-33.2 | 5.6 |
|  |  | 1996 | 2129 | 9.7 | 0-33.6 | 5.5 |
|  |  | 1997 | 2246 | 9.5 | 0-33.9 | 5.4 |
|  |  | 1998 | 2167 | 9.5 | 0-33.6 | 5.4 |
|  |  | 1999 | 1908 | 9.5 | 0-33.7 | 5.4 |
|  |  | 2000 | 2008 | 9.8 | 0-33.9 | 5.5 |
|  |  | 2001 | 2283 | 9.8 | 0-33.9 | 5.4 |
|  |  | 2002 | 2371 | 10.1 | 0-36.0 | 5.6 |
| Month | January | 1994 | 525 | 9.5 | 0-29.9 | 5.4 |
|  |  | 1995 | 670 | 9.2 | 0-31.0 | 5.3 |
|  |  | 1996 | 640 | 10.1 | 0-32.6 | 5.6 |
|  |  | 1997 | 746 | 10.0 | 0-33.3 | 5.6 |
|  |  | 1998 | 771 | 9.3 | 0-32.3 | 5.3 |
|  |  | 1999 | 565 | 9.7 | 0-32.3 | 5.5 |
|  |  | 2000 | 687 | 9.7 | 0-33.5 | 5.6 |
|  |  | 2001 | 704 | 9.9 | 0-33.7 | 5.5 |
|  |  | 2002 | 787 | 10.4 | 0-34.8 | 5.7 |
|  | February | 1994 | 565 | 8.9 | 0-32.1 | 5.1 |
|  |  | 1995 | 692 | 9.8 | 0-33.2 | 5.8 |
|  |  | 1996 | 654 | 9.7 | 0-31.9 | 5.5 |
|  |  | 1997 | 635 | 9.7 | 0-33.9 | 5.4 |
|  |  | 1998 | 620 | 9.9 | 0-33.1 | 5.5 |
|  |  | 1999 | 496 | 9.8 | 0-32.2 | 5.6 |
|  |  | 2000 | 575 | 9.9 | 0-33.5 | 5.6 |
|  |  | 2001 | 613 | 9.7 | 0-33.6 | 5.4 |
|  |  | 2002 | 688 | 10.2 | 0-33.9 | 5.6 |
|  | March | 1994 | 647 | 9.9 | 0-32.7 | 5.5 |
|  |  | 1995 | 798 | 9.3 | 0-32.7 | 5.5 |
|  |  | 1996 | 671 | 9.7 | 0-33.8 | 5.6 |
|  |  | 1997 | 679 | 9.7 | 0-33.1 | 5.4 |
|  |  | 1998 | 740 | 9.5 | 0-33.1 | 5.4 |
|  |  | 1999 | 618 | 9.5 | 0-32.6 | 5.3 |
|  |  | 2000 | 641 | 9.9 | 0-33.5 | 5.5 |
|  |  | 2001 | 757 | 9.5 | 0-32.8 | 5.4 |
|  |  | 2002 | 721 | 10.2 | 0-32.3 | 5.6 |
|  | April | 1994 | 645 | 9.4 | 0-30.8 | 5.4 |
|  |  | 1995 | 609 | 9.6 | 0-33.3 | 5.5 |
|  |  | 1996 | 679 | 9.6 | 0-32.7 | 5.5 |
|  |  | 1997 | 563 | 9.9 | 0-32.0 | 5.5 |
|  |  | 1998 | 660 | 9.1 | 0-31.1 | 5.2 |
|  |  | 1999 | 718 | 9.6 | 0-32.1 | 5.4 |
|  |  | 2000 | 716 | 9.3 | 0-31.6 | 5.2 |
|  |  | 2001 | 652 | 9.7 | 0-32.6 | 5.4 |
|  |  | 2002 | 722 | 10.3 | 0-32.9 | 5.7 |
|  | May | 1994 | 745 | 9.4 | 0-33.5 | 5.4 |
|  |  | 1995 | 643 | 9.6 | 0-33.2 | 5.6 |
|  |  | 1996 | 699 | 9.5 | 0-33.3 | 5.5 |
|  |  | 1997 | 702 | 9.3 | 0-33.0 | 5.2 |
|  |  | 1998 | 766 | 9.7 | 0-33.0 | 5.4 |
|  |  | 1999 | 787 | 9.9 | 0-33.2 | 5.6 |
|  |  | 2000 | 789 | 9.8 | 0-32.6 | 5.5 |
|  |  | 2001 | 869 | 9.4 | 0-33.2 | 5.2 |
|  |  | 2002 | 868 | 10.7 | 0-33.1 | 5.9 |
|  | June | 1994 | 781 | 9.1 | 0-31.8 | 5.3 |
|  |  | 1995 | 762 | 9.8 | 0-34.2 | 5.6 |
|  |  | 1996 | 746 | 9.6 | 0-33.6 | 5.5 |
|  |  | 1997 | 702 | 9.7 | 0-32.4 | 5.5 |
|  |  | 1998 | 766 | 9.7 | 0-33.2 | 5.5 |
|  |  | 1999 | 801 | 9.8 | 0-32.8 | 5.5 |
|  |  | 2000 | 812 | 9.7 | 0-33.2 | 5.5 |
|  |  | 2001 | 805 | 10.0 | 0-33.6 | 5.5 |
|  |  | 2002 | 802 | 9.5 | 0-32.5 | 5.5 |
|  | July | 1994 | 913 | 8.7 | 0-32.8 | 5.2 |
|  |  | 1995 | 820 | 9.3 | 0-34.1 | 5.6 |
|  |  | 1996 | 806 | 9.7 | 0-33.0 | 5.4 |
|  |  | 1997 | 902 | 9.3 | 0-33.7 | 5.4 |
|  |  | 1998 | 889 | 9.0 | 0-32.8 | 5.3 |
|  |  | 1999 | 849 | 9.9 | 0-33.7 | 5.5 |
|  |  | 2000 | 810 | 8.9 | 0-33.8 | 5.3 |
|  |  | 2001 | 975 | 9.2 | 0-33.9 | 5.3 |
|  |  | 2002 | 902 | 9.6 | 0-32.9 | 5.5 |
|  | August | 1994 | 828 | 8.6 | 0-32.3 | 5.2 |
|  |  | 1995 | 911 | 9.6 | 0-33.1 | 5.6 |
|  |  | 1996 | 863 | 9.4 | 0-33.2 | 5.4 |
|  |  | 1997 | 868 | 9.9 | 0-33.6 | 5.6 |
|  |  | 1998 | 978 | 8.7 | 0-34.1 | 5.1 |
|  |  | 1999 | 826 | 9.8 | 0-32.9 | 5.5 |
|  |  | 2000 | 786 | 9.6 | 0-33.6 | 5.4 |
|  |  | 2001 | 881 | 9.7 | 0-32.8 | 5.4 |
|  |  | 2002 | 941 | 9.8 | 0-32.7 | 5.5 |
|  | September | 1994 | 787 | 8.5 | 0-33.6 | 5.1 |
|  |  | 1995 | 755 | 9.2 | 0-32.3 | 5.3 |
|  |  | 1996 | 753 | 9.7 | 0-31.8 | 5.4 |
|  |  | 1997 | 777 | 9.6 | 0-32.9 | 5.4 |
|  |  | 1998 | 764 | 9.4 | 0-33.6 | 5.4 |
|  |  | 1999 | 783 | 9.4 | 0-33.6 | 5.3 |
|  |  | 2000 | 730 | 9.5 | 0-32.3 | 5.4 |
|  |  | 2001 | 821 | 9.8 | 0-33.5 | 5.4 |
|  |  | 2002 | 859 | 10.2 | 0-32.3 | 5.7 |
|  | October | 1994 | 812 | 9.1 | 0-32.4 | 5.1 |
|  |  | 1995 | 684 | 9.8 | 0-32.9 | 5.6 |
|  |  | 1996 | 741 | 9.6 | 0-30.7 | 5.3 |
|  |  | 1997 | 809 | 9.3 | 0-33.7 | 5.3 |
|  |  | 1998 | 760 | 10.0 | 0-34.0 | 5.6 |
|  |  | 1999 | 810 | 9.5 | 0-32.8 | 5.4 |
|  |  | 2000 | 687 | 9.5 | 0-31.3 | 5.4 |
|  |  | 2001 | 886 | 9.2 | 0-33.5 | 5.2 |
|  |  | 2002 | 841 | 10.3 | 0-33.4 | 5.7 |
|  | November | 1994 | 823 | 9.1 | 0-33.2 | 5.2 |
|  |  | 1995 | 747 | 10.0 | 0-33.3 | 5.6 |
|  |  | 1996 | 659 | 9.9 | 0-30.8 | 5.5 |
|  |  | 1997 | 705 | 9.1 | 0-33.0 | 5.3 |
|  |  | 1998 | 722 | 10.2 | 0-33.5 | 5.7 |
|  |  | 1999 | 753 | 9.4 | 0-33.1 | 5.3 |
|  |  | 2000 | 667 | 9.8 | 0-32.7 | 5.5 |
|  |  | 2001 | 894 | 9.6 | 0-32.7 | 5.3 |
|  |  | 2002 | 854 | 10.0 | 0-33.0 | 5.6 |
|  | December | 1994 | 792 | 8.8 | 0-32.0 | 5.2 |
|  |  | 1995 | 728 | 9.7 | 0-32.3 | 5.4 |
|  |  | 1996 | 766 | 9.4 | 0-33.5 | 5.4 |
|  |  | 1997 | 779 | 8.9 | 0-32.3 | 5.1 |
|  |  | 1998 | 692 | 9.5 | 0-33.3 | 5.5 |
|  |  | 1999 | 768 | 9.3 | 0-32.8 | 5.3 |
|  |  | 2000 | 672 | 9.8 | 0-32.4 | 5.4 |
|  |  | 2001 | 853 | 10.0 | 0-32.5 | 5.5 |
|  |  | 2002 | 811 | 9.9 | 0-33.1 | 5.5 |
| Workday | Weekday | 1994 | 6640 | 9.1 | 0-33.9 | 5.3 |
|  |  | 1995 | 6634 | 9.7 | 0-34.2 | 5.6 |
|  |  | 1996 | 6513 | 9.7 | 0-34.1 | 5.5 |
|  |  | 1997 | 6833 | 9.7 | 0-34.1 | 5.5 |
|  |  | 1998 | 6941 | 9.6 | 0-34.1 | 5.4 |
|  |  | 1999 | 6606 | 9.7 | 0-34.1 | 5.4 |
|  |  | 2000 | 6399 | 9.7 | 0-33.9 | 5.5 |
|  |  | 2001 | 7377 | 9.7 | 0-34.0 | 5.4 |
|  |  | 2002 | 7540 | 10.3 | 0-36.0 | 5.7 |
|  | Weekend | 1994 | 2223 | 8.9 | 0-33.0 | 5.2 |
|  |  | 1995 | 2185 | 9.2 | 0-34.1 | 5.4 |
|  |  | 1996 | 2164 | 9.5 | 0-34.0 | 5.4 |
|  |  | 1997 | 2034 | 9.0 | 0-32.7 | 5.2 |
|  |  | 1998 | 2187 | 9.3 | 0-34.0 | 5.4 |
|  |  | 1999 | 2168 | 9.5 | 0-33.8 | 5.5 |
|  |  | 2000 | 2173 | 9.3 | 0-33.4 | 5.3 |
|  |  | 2001 | 2333 | 9.4 | 0-33.9 | 5.3 |
|  |  | 2002 | 2256 | 9.6 | 0-33.3 | 5.4 |
| Day | Monday | 1994 | 1278 | 9.4 | 0-33.8 | 5.4 |
|  |  | 1995 | 1267 | 9.5 | 0-34.2 | 5.5 |
|  |  | 1996 | 1278 | 9.9 | 0-34.0 | 5.7 |
|  |  | 1997 | 1343 | 9.9 | 0-34.0 | 5.6 |
|  |  | 1998 | 1281 | 9.5 | 0-33.2 | 5.4 |
|  |  | 1999 | 1311 | 9.8 | 0-33.1 | 5.5 |
|  |  | 2000 | 1210 | 9.7 | 0-33.7 | 5.4 |
|  |  | 2001 | 1402 | 9.7 | 0-33.5 | 5.4 |
|  |  | 2002 | 1464 | 10.2 | 0-33.3 | 5.7 |
|  | Tuesday | 1994 | 1319 | 8.9 | 0-33.7 | 5.2 |
|  |  | 1995 | 1269 | 9.5 | 0-33.1 | 5.5 |
|  |  | 1996 | 1306 | 9.6 | 0-33.4 | 5.4 |
|  |  | 1997 | 1326 | 9.6 | 0-33.9 | 5.4 |
|  |  | 1998 | 1355 | 9.6 | 0-34.1 | 5.5 |
|  |  | 1999 | 1276 | 9.7 | 0-32.6 | 5.4 |
|  |  | 2000 | 1306 | 9.8 | 0-33.9 | 5.5 |
|  |  | 2001 | 1390 | 9.8 | 0-33.7 | 5.4 |
|  |  | 2002 | 1474 | 10.3 | 0-36.0 | 5.8 |
|  | Wednesday | 1994 | 1248 | 9.1 | 0-32.7 | 5.2 |
|  |  | 1995 | 1242 | 9.7 | 0-33.4 | 5.6 |
|  |  | 1996 | 1275 | 9.7 | 0-33.0 | 5.5 |
|  |  | 1997 | 1317 | 9.8 | 0-33.9 | 5.5 |
|  |  | 1998 | 1379 | 9.5 | 0-33.8 | 5.4 |
|  |  | 1999 | 1287 | 9.9 | 0-33.8 | 5.5 |
|  |  | 2000 | 1235 | 9.7 | 0-33.4 | 5.4 |
|  |  | 2001 | 1464 | 9.8 | 0-32.8 | 5.4 |
|  |  | 2002 | 1473 | 10.3 | 0-33.7 | 5.7 |
|  | Thursday | 1994 | 1257 | 9.0 | 0-32.8 | 5.2 |
|  |  | 1995 | 1348 | 10.0 | 0-33.4 | 5.7 |
|  |  | 1996 | 1264 | 9.7 | 0-33.0 | 5.4 |
|  |  | 1997 | 1362 | 9.8 | 0-33.4 | 5.5 |
|  |  | 1998 | 1395 | 9.4 | 0-32.8 | 5.3 |
|  |  | 1999 | 1304 | 9.5 | 0-33.3 | 5.4 |
|  |  | 2000 | 1282 | 9.7 | 0-33.6 | 5.5 |
|  |  | 2001 | 1448 | 9.6 | 0-33.7 | 5.3 |
|  |  | 2002 | 1514 | 10.3 | 0-33.3 | 5.7 |
|  | Friday | 1994 | 1538 | 9.3 | 0-32.8 | 5.3 |
|  |  | 1995 | 1508 | 9.7 | 0-33.2 | 5.6 |
|  |  | 1996 | 1390 | 9.6 | 0-33.1 | 5.4 |
|  |  | 1997 | 1485 | 9.4 | 0-33.6 | 5.3 |
|  |  | 1998 | 1531 | 9.7 | 0-33.8 | 5.5 |
|  |  | 1999 | 1428 | 9.6 | 0-33.2 | 5.4 |
|  |  | 2000 | 1366 | 9.7 | 0-33.3 | 5.5 |
|  |  | 2001 | 1673 | 9.7 | 0-33.9 | 5.4 |
|  |  | 2002 | 1615 | 10.1 | 0-32.8 | 5.6 |
|  | Saturday | 1994 | 1210 | 8.9 | 0-32.4 | 5.2 |
|  |  | 1995 | 1210 | 9.3 | 0-33.3 | 5.5 |
|  |  | 1996 | 1181 | 9.6 | 0-32.7 | 5.4 |
|  |  | 1997 | 1153 | 9.0 | 0-32.7 | 5.2 |
|  |  | 1998 | 1139 | 9.2 | 0-34.0 | 5.4 |
|  |  | 1999 | 1138 | 9.6 | 0-33.5 | 5.4 |
|  |  | 2000 | 1163 | 9.3 | 0-32.7 | 5.3 |
|  |  | 2001 | 1297 | 9.3 | 0-32.9 | 5.3 |
|  |  | 2002 | 1214 | 9.6 | 0-33.3 | 5.4 |
|  | Sunday | 1994 | 1013 | 8.9 | 0-32.9 | 5.2 |
|  |  | 1995 | 975 | 9.2 | 0-33.4 | 5.4 |
|  |  | 1996 | 983 | 9.4 | 0-34.0 | 5.4 |
|  |  | 1997 | 881 | 8.8 | 0-32.7 | 5.1 |
|  |  | 1998 | 1048 | 9.4 | 0-33.3 | 5.4 |
|  |  | 1999 | 1030 | 9.4 | 0-33.8 | 5.5 |
|  |  | 2000 | 1010 | 9.4 | 0-33.4 | 5.3 |
|  |  | 2001 | 1036 | 9.4 | 0-33.9 | 5.3 |
|  |  | 2002 | 1042 | 9.5 | 0-32.5 | 5.4 |
| All | Grand Mean |  | 1735 | 9.6 | 0-33.2 | 5.4 |
|  | Grand Min. |  | 496 | 8.5 | 0-29.9 | 5.1 |
|  | Grand Max. |  | 9796 | 10.7 | 0-36.0 | 5.9 |
|  | Grand Sd. |  | 1919 | 0.36 | 0-0.8 | 0.1 |
