# Supplementary material for: Identifying a spatial scale for the analysis of residential burglary: An empirical framework based on point pattern analysis
Source: PLoS One. 2022 Feb 28;17(2):e0264718. doi: 10.1371/journal.pone.0264718 (PMC8884495; doi:10.1371/journal.pone.0264718)
Supplement: S2 Table — (DOCX) [file pone.0264718.s002.docx]

**Table S2** **Data for** **Fig 3:** *L*_i_ (the thick black line) is calculated at each of the *h_i_* distances, *t_i_* is the theoretical *L* function value (null model) at *h_i_* (the red dashed line in Fig 3), *l_i_* and *u_i_* are the lower and upper boundaries of the simulation envelope (shaded area in Fig 3); The significance column indicates if *L_i_* is significantly (and consistently) higher than the upper boundary (*u_i_*) of the simulation envelope; the characteristic scale of clustering (*h_c_*) is calculated based on the *L_i_* and *h_i_* values where *L_i_* is significantly and consistently higher than *u_i_*, i.e., rows where significance column is ‘Y’.

| ***h_i_*** | ***L_i_*** | ***t*_i_** | ***l_i_*** | ***u_i_*** | **significance** |
| --- | --- | --- | --- | --- | --- |
| 43.3 | 145.3 | 10.5 | -36.6 | 121.0 | N |
| 57.7 | 145.1 | 19.4 | -24.4 | 113.1 | N |
| 72.2 | 149.0 | 18.4 | -34.3 | 108.8 | N |
| 86.6 | 159.9 | 24.7 | -35.3 | 165.8 | N |
| 101.0 | 151.3 | 20.5 | -42.6 | 156.7 | N |
| 115.5 | 149.4 | 23.5 | -45.3 | 149.8 | N |
| 129.9 | 145.0 | 25.7 | -45.9 | 139.2 | N |
| 144.3 | 151.9 | 24.6 | -53.2 | 134.1 | N |
| 158.8 | 154.5 | 32.2 | -40.0 | 158.7 | N |
| 173.2 | 150.9 | 31.0 | -47.1 | 150.2 | N |
| 187.6 | 147.5 | 35.9 | -37.4 | 158.1 | N |
| 202.0 | 146.0 | 34.8 | -36.7 | 153.8 | N |
| 216.5 | 142.2 | 34.9 | -42.9 | 144.7 | N |
| 230.9 | 150.7 | 37.8 | -51.0 | 177.7 | N |
| 245.3 | 147.3 | 35.0 | -52.9 | 172.5 | N |
| 259.8 | 145.5 | 32.7 | -62.8 | 197.0 | N |
| 274.2 | 143.9 | 30.7 | -64.0 | 200.3 | N |
| 288.6 | 143.6 | 29.3 | -53.0 | 188.8 | N |
| 303.1 | 132.5 | 33.7 | -42.8 | 179.5 | N |
| 317.5 | 151.8 | 38.4 | -46.2 | 292.4 | N |
| 331.9 | 152.2 | 41.0 | -48.2 | 282.6 | N |
| 346.4 | 162.6 | 39.4 | -67.1 | 278.7 | N |
| 360.8 | 208.8 | 40.0 | -40.5 | 270.1 | N |
| 375.2 | 223.2 | 46.2 | -67.4 | 262.6 | N |
| 389.7 | 221.4 | 43.4 | -68.5 | 253.1 | N |
| 404.1 | 229.9 | 43.1 | -71.9 | 247.9 | N |
| 418.5 | 228.1 | 52.2 | -59.2 | 245.3 | N |
| 433.0 | 228.0 | 48.0 | -49.2 | 232.9 | N |
| 447.4 | 226.5 | 50.6 | -52.6 | 225.5 | N |
| 461.8 | 226.7 | 52.6 | -50.1 | 243.7 | N |
| 476.3 | 229.1 | 52.3 | -45.6 | 240.2 | N |
| 490.7 | 239.0 | 57.6 | -48.9 | 233.8 | N |
| 505.1 | 233.6 | 52.5 | -48.9 | 235.5 | N |
| 519.5 | 295.3 | 53.4 | -56.8 | 225.5 | Y |
| 534.0 | 312.9 | 53.2 | -56.9 | 217.0 | Y |
| 548.4 | 310.6 | 58.7 | -61.3 | 262.2 | Y |
| 562.8 | 307.5 | 63.6 | -60.4 | 270.9 | Y |
| 577.3 | 329.1 | 61.4 | -48.6 | 262.5 | Y |
| 591.7 | 325.0 | 63.3 | -54.9 | 261.6 | Y |
| 606.1 | 328.0 | 63.1 | -57.7 | 253.7 | Y |
| 620.6 | 323.3 | 62.8 | -63.2 | 248.7 | Y |
| 635.0 | 320.3 | 61.7 | -70.0 | 244.0 | Y |
| 649.4 | 314.4 | 60.8 | -68.7 | 246.1 | Y |
| 663.9 | 311.1 | 58.2 | -74.3 | 241.1 | Y |
| 678.3 | 308.4 | 57.1 | -68.5 | 257.0 | Y |
| 692.7 | 312.7 | 55.3 | -66.8 | 257.4 | Y |
| 707.2 | 313.9 | 55.8 | -69.4 | 274.4 | Y |
| 721.6 | 309.8 | 54.4 | -60.4 | 272.3 | Y |
| 736.0 | 343.0 | 47.0 | -67.8 | 211.0 | Y |
| 750.5 | 347.2 | 42.1 | -69.8 | 232.9 | Y |
| 764.9 | 365.5 | 42.8 | -61.7 | 224.9 | Y |
| 779.3 | 364.3 | 42.0 | -45.6 | 238.0 | Y |
| 793.8 | 358.6 | 42.0 | -52.2 | 233.7 | Y |
| 808.2 | 358.5 | 40.7 | -53.8 | 223.0 | Y |
| 822.6 | 360.3 | 39.4 | -62.3 | 218.7 | Y |
| 837.0 | 353.1 | 36.6 | -72.1 | 235.1 | Y |
| 851.5 | 350.1 | 35.4 | -61.5 | 232.4 | Y |
| 865.9 | 358.1 | 35.0 | -57.5 | 227.8 | Y |
| 880.3 | 389.3 | 32.7 | -62.5 | 218.6 | Y |
| 894.8 | 387.7 | 28.4 | -55.2 | 212.2 | Y |
| 909.2 | 392.4 | 28.0 | -52.0 | 206.7 | Y |
| 923.6 | 386.2 | 29.1 | -51.5 | 206.1 | Y |
| 938.1 | 397.0 | 27.3 | -52.2 | 200.9 | Y |
| 952.5 | 411.0 | 26.7 | -55.6 | 192.6 | Y |
| 966.9 | 405.9 | 25.3 | -53.4 | 194.5 | Y |
| 981.4 | 406.4 | 26.0 | -51.7 | 193.8 | Y |
| 995.8 | 414.4 | 24.8 | -50.2 | 182.3 | Y |
| 1010.2 | 413.1 | 23.0 | -44.9 | 173.9 | Y |
| 1024.7 | 417.6 | 21.2 | -47.6 | 144.3 | Y |
| 1039.1 | 447.7 | 20.6 | -50.2 | 142.2 | Y |
| 1053.5 | 444.9 | 21.5 | -48.1 | 147.4 | Y |
| 1068.0 | 452.4 | 21.0 | -53.2 | 145.9 | Y |
| 1082.4 | 452.8 | 21.3 | -56.3 | 143.9 | Y |
| 1096.8 | 460.2 | 22.9 | -57.0 | 138.4 | Y |
| 1111.3 | 561.3 | 20.4 | -64.1 | 134.0 | Y |
| 1125.7 | 569.9 | 20.2 | -68.2 | 140.0 | Y |
| 1140.1 | 568.4 | 22.5 | -67.5 | 149.2 | Y |
| 1154.5 | 569.2 | 20.2 | -69.3 | 151.5 | Y |
| 1169.0 | 570.6 | 23.8 | -72.8 | 148.6 | Y |
| 1183.4 | 568.0 | 27.3 | -70.1 | 184.2 | Y |
| 1197.8 | 567.4 | 28.5 | -61.7 | 183.9 | Y |
| 1212.3 | 576.3 | 29.4 | -70.9 | 195.2 | Y |
| 1226.7 | 572.0 | 30.1 | -84.8 | 239.8 | Y |
| 1241.1 | 603.2 | 27.3 | -87.0 | 230.6 | Y |
| 1255.6 | 638.4 | 32.4 | -86.7 | 230.4 | Y |
| 1270.0 | 640.0 | 32.8 | -94.2 | 244.9 | Y |
| 1284.4 | 636.6 | 32.2 | -89.8 | 251.0 | Y |
| 1298.9 | 637.1 | 32.2 | -96.3 | 292.8 | Y |
| 1313.3 | 633.4 | 31.1 | -95.3 | 296.2 | Y |
| 1327.7 | 631.9 | 31.1 | -103.4 | 295.8 | Y |
| 1342.2 | 628.0 | 29.7 | -109.8 | 300.9 | Y |
| 1356.6 | 621.4 | 29.0 | -106.2 | 290.4 | Y |
| 1371.0 | 624.1 | 28.3 | -106.0 | 293.0 | Y |
| 1385.5 | 642.3 | 30.5 | -105.5 | 293.2 | Y |
| 1399.9 | 641.6 | 30.6 | -111.6 | 294.5 | Y |
| 1414.3 | 641.5 | 30.8 | -113.5 | 291.2 | Y |
| 1428.8 | 700.9 | 30.3 | -112.2 | 293.2 | Y |
| 1443.2 | 699.8 | 30.7 | -116.4 | 292.0 | Y |
| 1457.6 | 717.8 | 32.9 | -115.6 | 292.1 | Y |
| 1472.0 | 718.3 | 33.0 | -100.0 | 294.3 | Y |
| 1486.5 | 717.3 | 33.5 | -106.6 | 297.2 | Y |
| 1500.9 | 716.5 | 33.9 | -113.1 | 317.8 | Y |
| 1515.3 | 723.7 | 34.4 | -121.0 | 330.0 | Y |
| 1529.8 | 741.8 | 34.1 | -110.3 | 327.2 | Y |
| 1544.2 | 741.0 | 33.8 | -112.9 | 325.5 | Y |
| 1558.6 | 739.8 | 35.0 | -118.2 | 321.8 | Y |
| 1573.1 | 735.7 | 34.4 | -121.5 | 325.2 | Y |
| 1587.5 | 734.5 | 34.4 | -122.1 | 327.8 | Y |
| 1601.9 | 726.2 | 34.2 | -110.4 | 321.4 | Y |
| 1616.4 | 717.8 | 35.1 | -115.7 | 323.8 | Y |
| 1630.8 | 726.3 | 39.2 | -118.2 | 331.4 | Y |
| 1645.2 | 741.5 | 39.9 | -110.4 | 337.4 | Y |
| 1659.7 | 745.4 | 41.7 | -85.3 | 331.1 | Y |
| 1674.1 | 747.9 | 42.3 | -91.9 | 337.0 | Y |
| 1688.5 | 755.8 | 42.9 | -95.2 | 351.4 | Y |
| 1703.0 | 752.2 | 46.0 | -88.8 | 347.4 | Y |
| 1717.4 | 773.2 | 46.4 | -97.9 | 355.6 | Y |
| 1731.8 | 801.9 | 47.1 | -103.8 | 357.9 | Y |
| 1746.3 | 795.0 | 47.6 | -105.6 | 363.3 | Y |
| 1760.7 | 807.5 | 51.1 | -109.9 | 379.0 | Y |
| 1775.1 | 815.3 | 52.5 | -115.2 | 402.7 | Y |
| 1789.5 | 816.6 | 55.6 | -117.1 | 396.3 | Y |
| 1804.0 | 823.0 | 57.4 | -119.1 | 389.8 | Y |
| 1818.4 | 821.3 | 58.7 | -117.8 | 390.2 | Y |
| 1832.8 | 828.6 | 59.2 | -118.3 | 387.9 | Y |
| 1847.3 | 821.2 | 61.0 | -121.1 | 384.3 | Y |
| 1861.7 | 815.5 | 62.4 | -125.2 | 380.6 | Y |
| 1876.1 | 812.9 | 63.4 | -125.1 | 381.7 | Y |
| 1890.6 | 806.4 | 63.2 | -124.3 | 401.1 | Y |
| 1905.0 | 816.6 | 63.4 | -129.4 | 408.7 | Y |
| 1919.4 | 827.9 | 63.9 | -129.2 | 404.7 | Y |
| 1933.9 | 834.0 | 62.1 | -134.0 | 401.1 | Y |
| 1948.3 | 839.0 | 64.1 | -120.5 | 398.1 | Y |
| 1962.7 | 839.1 | 67.2 | -121.2 | 393.2 | Y |
| 1977.2 | 839.4 | 68.7 | -115.5 | 390.0 | Y |
| 1991.6 | 844.9 | 68.4 | -119.5 | 388.7 | Y |
| 2006.0 | 845.4 | 69.9 | -118.7 | 389.9 | Y |
| 2020.5 | 833.0 | 71.9 | -115.6 | 388.9 | Y |
| 2034.9 | 827.9 | 70.4 | -115.7 | 393.3 | Y |
| 2049.3 | 834.7 | 72.5 | -116.7 | 405.9 | Y |
| 2063.8 | 834.7 | 74.1 | -123.2 | 413.5 | Y |
| 2078.2 | 832.1 | 75.8 | -121.9 | 411.7 | Y |
| 2092.6 | 834.9 | 75.5 | -122.5 | 407.6 | Y |
| 2107.0 | 845.2 | 76.6 | -129.5 | 402.6 | Y |
| 2121.5 | 854.6 | 78.0 | -114.6 | 408.5 | Y |
| 2135.9 | 856.5 | 78.7 | -115.1 | 403.8 | Y |
| 2150.3 | 872.9 | 80.1 | -112.5 | 407.4 | Y |
| 2164.8 | 864.7 | 82.4 | -116.4 | 408.2 | Y |
| 2179.2 | 864.2 | 81.6 | -122.2 | 420.1 | Y |
| 2193.6 | 862.4 | 83.0 | -127.2 | 429.2 | Y |
| 2208.1 | 852.5 | 83.3 | -130.8 | 428.8 | Y |
| 2222.5 | 847.0 | 83.7 | -130.8 | 440.1 | Y |
| 2236.9 | 842.8 | 84.6 | -134.8 | 437.9 | Y |
| 2251.4 | 854.3 | 86.3 | -139.5 | 434.0 | Y |
| 2265.8 | 858.5 | 86.3 | -143.1 | 447.5 | Y |
| 2280.2 | 855.4 | 86.6 | -145.6 | 450.3 | Y |
| 2294.7 | 889.0 | 87.2 | -146.0 | 457.3 | Y |
| 2309.1 | 884.4 | 89.3 | -147.0 | 477.3 | Y |
| 2323.5 | 891.0 | 90.8 | -147.6 | 477.9 | Y |
| 2338.0 | 880.9 | 91.1 | -146.4 | 476.0 | Y |
| 2352.4 | 876.3 | 91.1 | -150.1 | 470.1 | Y |
| 2366.8 | 874.1 | 91.6 | -159.0 | 462.7 | Y |
| 2381.3 | 881.3 | 92.8 | -161.7 | 462.1 | Y |
| 2395.7 | 874.1 | 92.2 | -159.4 | 436.3 | Y |
| 2410.1 | 863.5 | 92.8 | -145.1 | 438.0 | Y |
| 2424.6 | 858.3 | 92.3 | -145.0 | 445.1 | Y |
| 2439.0 | 853.0 | 94.0 | -150.3 | 447.5 | Y |
| 2453.4 | 848.5 | 94.0 | -148.1 | 448.0 | Y |
| 2467.8 | 872.6 | 94.0 | -144.0 | 425.4 | Y |
| 2482.3 | 869.9 | 95.7 | -142.6 | 453.5 | Y |
| 2496.7 | 866.0 | 96.1 | -126.6 | 452.1 | Y |
| 2511.1 | 860.3 | 98.4 | -128.9 | 476.5 | Y |
| 2525.6 | 850.2 | 96.6 | -126.2 | 472.9 | Y |
| 2540.0 | 837.6 | 96.9 | -117.6 | 476.5 | Y |
| 2554.4 | 853.1 | 95.7 | -113.4 | 486.1 | Y |
| 2568.9 | 891.2 | 96.6 | -118.5 | 481.7 | Y |
| 2583.3 | 884.5 | 96.2 | -125.3 | 483.0 | Y |
| 2597.7 | 843.6 | 96.7 | -118.6 | 463.0 | Y |
| 2612.2 | 830.3 | 97.9 | -127.2 | 478.1 | Y |
| 2626.6 | 843.2 | 96.2 | -132.1 | 491.0 | Y |
| 2641.0 | 881.4 | 97.4 | -136.2 | 500.6 | Y |
| 2655.5 | 904.0 | 98.5 | -139.6 | 499.6 | Y |
| 2669.9 | 898.9 | 99.8 | -144.0 | 507.4 | Y |
| 2684.3 | 864.7 | 99.8 | -144.9 | 502.7 | Y |
| 2698.8 | 860.9 | 101.8 | -149.5 | 502.3 | Y |
| 2713.2 | 854.6 | 104.0 | -150.3 | 501.4 | Y |
| 2727.6 | 868.3 | 105.6 | -154.3 | 497.5 | Y |
| 2742.1 | 868.0 | 105.2 | -152.6 | 502.9 | Y |
| 2756.5 | 873.0 | 106.8 | -149.1 | 507.4 | Y |
| 2770.9 | 873.5 | 108.5 | -153.6 | 534.6 | Y |
| 2785.3 | 867.0 | 107.3 | -159.5 | 541.7 | Y |
| 2799.8 | 853.6 | 109.6 | -163.9 | 545.4 | Y |
| 2814.2 | 852.7 | 110.9 | -159.6 | 558.3 | Y |
| 2828.6 | 876.4 | 113.5 | -161.0 | 563.4 | Y |
| 2843.1 | 874.2 | 113.9 | -166.7 | 583.1 | Y |
| 2857.5 | 865.3 | 113.2 | -166.8 | 575.1 | Y |
| 2871.9 | 861.1 | 115.1 | -161.3 | 571.7 | Y |
| 2886.4 | 853.3 | 117.0 | -169.1 | 575.7 | Y |
| 2900.8 | 847.4 | 116.9 | -184.3 | 581.8 | Y |
| 2915.2 | 843.9 | 116.3 | -188.4 | 585.8 | Y |
| 2929.7 | 779.8 | 115.6 | -185.7 | 578.2 | Y |
| 2944.1 | 792.7 | 113.2 | -195.2 | 580.4 | Y |
| 2958.5 | 800.2 | 114.8 | -189.3 | 595.7 | Y |
| 2973.0 | 792.9 | 114.9 | -200.3 | 592.0 | Y |
| 2987.4 | 787.9 | 113.9 | -214.6 | 595.9 | Y |
| 3001.8 | 779.3 | 115.9 | -214.8 | 592.8 | Y |
| 3016.3 | 789.8 | 116.4 | -195.5 | 588.5 | Y |
| 3030.7 | 789.6 | 114.7 | -198.1 | 598.8 | Y |
| 3045.1 | 795.0 | 115.5 | -222.8 | 597.8 | Y |
| 3059.6 | 805.3 | 118.1 | -208.1 | 601.7 | Y |
| 3074.0 | 809.4 | 120.4 | -214.0 | 606.8 | Y |
| 3088.4 | 818.8 | 118.7 | -217.4 | 603.1 | Y |
| 3102.8 | 733.7 | 119.3 | -219.7 | 602.2 | Y |
| 3117.3 | 737.9 | 117.6 | -222.6 | 596.1 | Y |
| 3131.7 | 734.3 | 118.2 | -251.7 | 624.1 | Y |
| 3146.1 | 734.7 | 117.4 | -268.3 | 616.9 | Y |
| 3160.6 | 724.4 | 121.5 | -268.5 | 633.3 | Y |
| 3175.0 | 735.9 | 121.6 | -265.6 | 631.6 | Y |
| 3189.4 | 725.7 | 124.5 | -270.9 | 673.8 | Y |
| 3203.9 | 727.1 | 123.3 | -274.3 | 680.6 | Y |
| 3218.3 | 722.6 | 123.6 | -268.5 | 675.1 | Y |
| 3232.7 | 715.9 | 120.4 | -274.2 | 678.8 | Y |
| 3247.2 | 715.6 | 121.3 | -269.4 | 684.2 | Y |
| 3261.6 | 708.5 | 122.4 | -278.5 | 709.8 | N |
| 3276.0 | 773.7 | 121.7 | -288.7 | 711.2 | N |
| 3290.5 | 768.6 | 122.4 | -292.2 | 726.5 | N |
| 3304.9 | 759.0 | 121.6 | -293.3 | 727.6 | N |
| 3319.3 | 787.7 | 124.1 | -304.0 | 740.2 | N |
| 3333.8 | 778.6 | 123.3 | -304.4 | 736.3 | N |
| 3348.2 | 774.9 | 121.4 | -308.2 | 747.5 | N |
| 3362.6 | 774.4 | 121.8 | -308.1 | 746.1 | N |
| 3377.1 | 794.0 | 121.7 | -307.3 | 762.2 | N |
| 3391.5 | 788.4 | 122.9 | -308.4 | 764.0 | N |
| 3405.9 | 790.5 | 123.6 | -306.0 | 767.7 | N |
| 3420.3 | 779.6 | 125.3 | -309.1 | 788.5 | N |
| 3434.8 | 770.1 | 125.3 | -318.5 | 787.0 | N |
| 3449.2 | 771.7 | 123.7 | -321.2 | 781.1 | N |
| 3463.6 | 766.7 | 126.2 | -324.5 | 787.7 | N |
| 3478.1 | 765.5 | 127.4 | -320.4 | 786.7 | N |
| 3492.5 | 761.0 | 126.1 | -323.6 | 793.8 | N |
| 3506.9 | 756.5 | 126.4 | -330.0 | 801.3 | N |
| 3521.4 | 752.7 | 127.4 | -335.1 | 800.0 | N |
| 3535.8 | 752.9 | 129.5 | -330.2 | 796.5 | N |
| 3550.2 | 748.6 | 128.8 | -332.5 | 796.7 | N |
| 3564.7 | 816.7 | 129.8 | -340.2 | 801.5 | N |
| 3579.1 | 814.6 | 129.7 | -344.6 | 793.5 | N |
| 3593.5 | 806.4 | 129.1 | -345.7 | 798.9 | N |
| 3608.0 | 798.1 | 129.7 | -339.2 | 796.5 | N |
| 3622.4 | 795.1 | 129.3 | -340.8 | 790.0 | N |
| 3636.8 | 791.0 | 130.3 | -344.8 | 804.0 | N |
| 3651.3 | 778.5 | 130.8 | -346.8 | 799.5 | N |
| 3665.7 | 773.4 | 132.3 | -350.7 | 805.0 | N |
| 3680.1 | 772.5 | 132.6 | -354.3 | 831.3 | N |
| 3694.6 | 764.9 | 134.5 | -351.3 | 832.2 | N |
| 3709.0 | 756.3 | 137.1 | -283.8 | 829.1 | N |
| 3723.4 | 748.2 | 136.8 | -279.8 | 830.6 | N |
| 3737.8 | 757.7 | 135.3 | -282.3 | 828.5 | N |
| 3752.3 | 747.0 | 138.5 | -287.4 | 843.6 | N |
| 3766.7 | 741.8 | 138.2 | -311.7 | 867.5 | N |
| 3781.1 | 782.1 | 138.7 | -306.6 | 866.3 | N |
| 3795.6 | 773.4 | 139.8 | -311.5 | 869.3 | N |
| 3810.0 | 764.6 | 141.0 | -305.5 | 875.6 | N |
| 3824.4 | 728.2 | 142.9 | -321.3 | 861.6 | N |
| 3838.9 | 723.3 | 146.2 | -328.2 | 885.5 | N |
| 3853.3 | 715.8 | 145.0 | -331.5 | 892.8 | N |
| 3867.7 | 695.2 | 147.1 | -322.4 | 896.4 | N |
| 3882.2 | 700.3 | 147.3 | -329.0 | 894.5 | N |
| 3896.6 | 687.9 | 147.4 | -329.0 | 897.2 | N |
| 3911.0 | 689.6 | 148.1 | -332.9 | 902.5 | N |
| 3925.5 | 688.8 | 147.8 | -333.6 | 900.9 | N |
| 3939.9 | 683.3 | 149.4 | -337.8 | 900.1 | N |
| 3954.3 | 677.4 | 151.5 | -334.8 | 905.4 | N |
| 3968.8 | 670.3 | 152.4 | -337.5 | 923.3 | N |
| 3983.2 | 672.2 | 152.7 | -330.5 | 927.3 | N |
| 3997.6 | 669.4 | 157.6 | -307.1 | 932.8 | N |
| 4012.1 | 666.1 | 157.9 | -309.1 | 942.4 | N |
| 4026.5 | 717.6 | 159.1 | -312.2 | 962.5 | N |
| 4040.9 | 710.6 | 159.9 | -300.8 | 963.7 | N |
| 4055.3 | 708.7 | 160.2 | -311.1 | 960.7 | N |
| 4069.8 | 701.0 | 158.4 | -316.2 | 959.3 | N |
| 4084.2 | 701.6 | 160.6 | -314.0 | 953.9 | N |
| 4098.6 | 696.2 | 161.7 | -324.9 | 952.8 | N |
| 4113.1 | 694.5 | 162.7 | -329.0 | 969.0 | N |
| 4127.5 | 681.1 | 162.3 | -347.5 | 967.8 | N |
| 4141.9 | 680.0 | 165.2 | -341.5 | 990.2 | N |
| 4156.4 | 671.4 | 164.1 | -334.4 | 991.2 | N |
| 4170.8 | 673.1 | 164.2 | -341.8 | 999.8 | N |
| 4185.2 | 655.7 | 165.6 | -332.2 | 1005.7 | N |
| 4199.7 | 653.3 | 166.4 | -332.4 | 1016.2 | N |
| 4214.1 | 643.6 | 167.0 | -347.5 | 1014.3 | N |
| 4228.5 | 637.9 | 167.7 | -349.3 | 1015.5 | N |
| 4243.0 | 641.3 | 168.0 | -336.9 | 1014.2 | N |
| 4257.4 | 633.4 | 169.2 | -337.8 | 1008.9 | N |
| 4271.8 | 629.9 | 168.3 | -340.1 | 1008.3 | N |
| 4286.3 | 621.3 | 168.9 | -340.5 | 1009.5 | N |
| 4300.7 | 614.2 | 173.0 | -321.6 | 1011.8 | N |
| 4315.1 | 604.0 | 173.8 | -328.5 | 1020.5 | N |
| 4329.6 | 593.4 | 172.4 | -332.1 | 1013.1 | N |
| 4344.0 | 590.3 | 175.8 | -327.6 | 1016.7 | N |
| 4358.4 | 578.8 | 175.0 | -331.2 | 1018.8 | N |
| 4372.8 | 578.6 | 176.8 | -346.3 | 1025.7 | N |
| 4387.3 | 569.3 | 175.8 | -350.1 | 1032.9 | N |
| 4401.7 | 572.4 | 175.2 | -342.1 | 1032.5 | N |
| 4416.1 | 690.4 | 175.2 | -351.8 | 1033.4 | N |
| 4430.6 | 680.9 | 174.4 | -350.9 | 1028.9 | N |
| 4445.0 | 675.2 | 174.3 | -342.1 | 1022.7 | N |
| 4459.4 | 662.8 | 175.1 | -357.5 | 1022.4 | N |
| 4473.9 | 653.1 | 177.2 | -360.7 | 1033.1 | N |
| 4488.3 | 651.5 | 174.7 | -366.5 | 1038.2 | N |
| 4502.7 | 644.2 | 173.0 | -375.1 | 1025.1 | N |
| 4517.2 | 635.1 | 171.0 | -377.4 | 1033.9 | N |
| 4531.6 | 626.1 | 169.2 | -378.3 | 1039.1 | N |
| 4546.0 | 616.3 | 172.3 | -383.1 | 1033.5 | N |
| 4560.5 | 610.3 | 171.5 | -387.3 | 1029.2 | N |
| 4574.9 | 608.4 | 176.1 | -388.4 | 1022.1 | N |
| 4589.3 | 597.1 | 175.6 | -395.4 | 1021.6 | N |
| 4603.8 | 585.7 | 178.6 | -395.2 | 1013.6 | N |
| 4618.2 | 576.2 | 183.1 | -383.6 | 1138.2 | N |
| 4632.6 | 567.9 | 181.7 | -392.2 | 1130.4 | N |
| 4647.1 | 563.2 | 180.6 | -354.5 | 1125.3 | N |
| 4661.5 | 563.2 | 176.7 | -356.1 | 1126.0 | N |
| 4675.9 | 556.5 | 175.4 | -377.6 | 1136.1 | N |
| 4690.3 | 555.3 | 172.7 | -384.0 | 1131.2 | N |
| 4704.8 | 557.1 | 172.9 | -383.4 | 1130.5 | N |
| 4719.2 | 549.0 | 170.7 | -374.7 | 1127.4 | N |
| 4733.6 | 560.1 | 167.1 | -377.6 | 1122.8 | N |
| 4748.1 | 555.0 | 168.1 | -372.8 | 1119.6 | N |
| 4762.5 | 567.1 | 165.1 | -378.7 | 1117.6 | N |
| 4776.9 | 556.0 | 164.5 | -372.6 | 1120.4 | N |
| 4791.4 | 544.6 | 171.8 | -381.4 | 1114.5 | N |
| 4805.8 | 538.0 | 168.4 | -377.9 | 1112.2 | N |
| 4820.2 | 539.4 | 165.0 | -371.0 | 1100.7 | N |
| 4834.7 | 528.1 | 161.3 | -361.3 | 1097.4 | N |
| 4849.1 | 516.9 | 157.5 | -376.2 | 1091.6 | N |
| 4863.5 | 518.3 | 156.5 | -399.3 | 1082.9 | N |
| 4878.0 | 519.5 | 151.8 | -433.3 | 1076.0 | N |
| 4892.4 | 514.4 | 150.3 | -424.2 | 1073.8 | N |
| 4906.8 | 504.8 | 148.6 | -425.5 | 1088.1 | N |
| 4921.3 | 520.4 | 140.6 | -474.9 | 1079.2 | N |
| 4935.7 | 509.8 | 137.7 | -480.1 | 1068.8 | N |
| 4950.1 | 498.7 | 137.3 | -487.2 | 1077.9 | N |
| 4964.6 | 495.7 | 130.6 | -575.0 | 1085.6 | N |
| 4979.0 | 516.8 | 128.4 | -586.5 | 1090.8 | N |
| 4993.4 | 515.3 | 134.2 | -595.9 | 1093.2 | N |
| 5007.9 | 506.5 | 130.3 | -596.0 | 1099.7 | N |
| 5022.3 | 504.9 | 128.7 | -609.5 | 1092.8 | N |
| 5036.7 | 513.3 | 126.6 | -617.8 | 1090.4 | N |
| 5051.1 | 505.6 | 125.3 | -578.2 | 1078.4 | N |
| 5065.6 | 506.0 | 124.1 | -640.1 | 1072.2 | N |
| 5080.0 | 502.6 | 122.3 | -650.2 | 1066.7 | N |
| 5094.4 | 491.5 | 117.6 | -647.6 | 1061.9 | N |
